# The bovine TRPV3 as a pathway for the uptake of Na^+^, Ca^2+^, and NH_4_^+^

**DOI:** 10.1371/journal.pone.0193519

**Published:** 2018-03-01

**Authors:** Katharina T. Schrapers, Gerhard Sponder, Franziska Liebe, Hendrik Liebe, Friederike Stumpff

**Affiliations:** Institute of Veterinary Physiology, Faculty of Veterinary Medicine, Freie Universität Berlin, Berlin, Germany; Indiana University School of Medicine, UNITED STATES

## Abstract

Absorption of ammonia from the gastrointestinal tract results in problems that range from hepatic encephalopathy in humans to poor nitrogen efficiency of cattle with consequences for the global climate. Previous studies on epithelia and cells from the native ruminal epithelium suggest functional involvement of the bovine homologue of TRPV3 (bTRPV3) in ruminal NH_4_^+^ transport. Since the conductance of TRP channels to NH_4_^+^ has never been studied, bTRPV3 was overexpressed in HEK-293 cells and investigated using the patch-clamp technique and intracellular calcium imaging. Control cells contained the empty construct. Divalent cations blocked the conductance for monovalent cations in both cell types, with effects higher in cells expressing bTRPV3. In bTRPV3 cells, but not in controls, menthol, thymol, carvacrol, or 2-APB stimulated whole cell currents mediated by Na^+^, Cs^+^, NH_4_^+^_,_ and K^+^, with a rise in intracellular Ca^2+^ observed in response to menthol. While only 25% of control patches showed single-channel events (with a conductance of 40.8 ± 11.9 pS for NH_4_^+^ and 25.0 ± 5.8 pS for Na^+^), 90% of bTRPV3 patches showed much larger conductances of 127.8 ± 4.2 pS for Na^+^, 240.1 ± 3.6 pS for NH_4_^+^, 34.0 ± 1.7 pS for Ca^2+^, and ~ 36 pS for NMDG^+^. Open probability, but not conductance, rose with time after patch excision. In conjunction with previous research, we suggest that bTRPV3 channels may play a role in the transport of Na^+^, K^+^, Ca^2+^ and NH_4_^+^ across the rumen with possible repercussions for understanding the function of TRPV3 in other epithelia.

## Introduction

The transient receptor potential (TRP) multigene superfamily consists of 28 known sequences that are subdivided into seven subfamilies, six of which are found in mammalian tissues and encode for integral membrane proteins that function as cation-selective channels in extra- or intracellular membranes [1, 2]. Possibly owing to the original discovery in the sensory system of drosophila flies [3], most functional research to date has focused on the role of TRP channels in Ca^2+^ mediated sensory signaling cascades, such as those involved in the detection of thermal signals, inflammation, or pain [4]. Intriguingly, the fragrant constituents found in herbs have been found to interact in a specific manner with various members of the TRP channel family, triggering an influx of Ca^2+^ and Na^+^ [5, 6]. Since TRP channels are expressed by almost every cell type of the body, this opens an exciting new field for pharmacological interventions that are currently being explored [2].

In addition to what is by now classical role in cellular signaling, a direct role in epithelial transport has been established for TRPV5 and TRPV6 in the renal and intestinal transport of Ca^2+^ [7, 8] with similar functions played by TRPM6 and TRPM7 in the transport of Mg^2+^ [9, 10]. However, there is currently very limited information regarding the functional role of TRP channels in the epithelial transport of monovalent cations and none concerning the physiologically important NH_4_^+^ ion.

Our own interest in TRP channels was sparked when attempting to identify proteins involved in ammonia transport by gastrointestinal epithelia [11–13]. Large quantities of ammonia are produced in the fermentative parts of the gut from microbial degradation of nitrogenous compounds and subsequently absorbed. Since ammonia is highly toxic, it has to be converted to urea or glutamine by the liver and excreted via the kidney [14], leading to multiple associated problems. In humans suffering from hepatic disease, high colonic absorption of ammonia worsens cerebral function [15]. In patients with kidney dysfunction, uremia is aggravated [16]. In livestock, absorption of ammonia from the gut decreases microbial protein synthesis while increasing nitrogen emissions with subsequent formation of climate gasses. The problems are formidable: cattle alone are accountable for about 30% of global nitrogen emissions or twice the contribution of all oceans combined [17, 18].

A closer look is thus warranted and shows that matters are more complex since not all urea that is generated from ammonia is renally excreted. Instead, variable quantities are recycled to the gut via proteins such as UT-B that are specifically expressed for the purpose [19]. In contrast to the host animal, the bacteria of the gut can utilize urea as a source of nitrogen for the generation of high-grade protein incorporating essential amino-acids, which, in the case of cattle, can be fully digested and utilized [20, 21]. However, further amounts of the secreted urea are again degraded to ammonia, reabsorbed, detoxified to urea, and re-secreted. This gastrointestinal recycling of nitrogen frequently exceeds 20 mol∙day^−1^ in dairy cows [22], and it is estimated that reconversion of ammonia to urea accounts for 10% of the total energy required by the liver of cattle. Gastrointestinal recycling of nitrogen is also found across hindgut epithelia of monogastric species such as the pig [12] or in humans [23]. It may be asked what purpose it serves. A potential reason emerges when considering that for each mole of urea secreted into the gut, two moles of the strong buffer NH_3_ are formed. Accordingly and provided that the subsequent absorption occurs in the protonated form as NH_4_^+^ through suitable transport proteins, gastrointestinal recycling of nitrogen might contribute to luminal pH homeostasis. Finally, if the transport proteins were known, it might be possible to pharmacologically modulate ammonia absorption from the gut. There are thus multiple reasons why a more detailed knowledge concerning the mechanisms behind the absorption of ammonia appears urgent.

It is by now well established that the high permeability of most cellular membranes to ammonia is linked to the expression of various more or less specific transport proteins [14]. Ruminal physiologists were among the first to challenge the notion of non-specific diffusion of the uncharged form (NH_3_) [24] and it has been shown that at physiological pH, transport across the rumen occurs primarily in the form of NH_4_^+^ via cation channels [11, 25]. Subsequently, further studies of the ruminal epithelium on the level of the tissue and the cell demonstrated that these channels are non-selective and sensitive both to divalent cations [13, 26, 27] and to the TRPV3 channel agonists menthol and thymol [13]. In conjunction with mRNA data from the native ruminal epithelium excluding TRPM8, the non-selective cation channel TRPV3 has emerged as a possible candidate mediating the transport of cations across the tissue. However, the permeability of TRP channels in general and of TRPV3 in particular to NH_4_^+^ has never been investigated [28]. The current study fills this gap.

On the basis of measurements in the whole cell and single-channel configuration of the patch-clamp technique, we report that the bovine representative of this channel has a conductance to NH_4_^+^ that exceeds that of Na^+^ and Ca^2+^. To our knowledge, this is the first time that the conductance of a homologue of TRPV3 to either Ca^2+^ or NH_4_^+^ has been studied on the single-channel level. Based on data from whole cell and single-channel experiments, we further report a marginal conductance to the organic cation NMDG^+^. The bTRPV3 may thus play an important role in mediating transport not just of Na^+^ or Ca^2+^, but also to NH_4_^+^ and other organic cations with possible repercussions for understanding the function of this poorly understood protein in health and disease [29].

## Material and methods

### Cloning of bTRPV3 into pIRES2-AcGFP1 and pcDNA5/TO

The bovine sequence of TRPV3 (XM_015458625.1) with an N-terminal HA-Strep-tag was produced by gene synthesis (ShineGene Bio-Technologies Inc., Shanghai, China). The dual tag was placed at the N-terminus in order to prevent possible interference with a C-terminal PDZ binding motif found in some TRP channels [1]. The HA-Strep-bTRPV3 construct was then subcloned into pIRES2-AcGFP1 (Clontech Laboratories, Mountain View, CA, USA) via NheI and EcoRI restriction sites and into pcDNA5/TO (Life Technologies, Darmstadt, Germany) via the restriction sites HindIII and KpnI.

### Cell culture and transient transfection of bTRPV3

**Fig 1:**
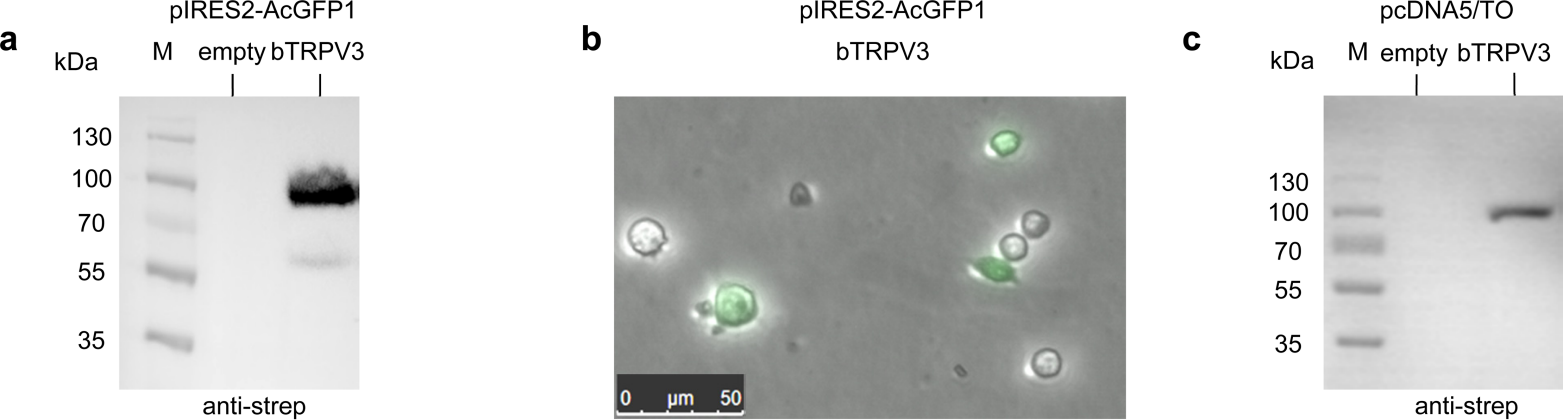
Immunoblots and fluorescence microscopy images of HEK-293 cells expressing bTRPV3. a) Immunoblot of HEK-293 cell lysates 24 hours after transfection with a pIRES2-AcGFP1 vector. The marker lane (M) is followed by a lane of control cells transfected with the empty pIRES2-AcGFP1 vector (no band), followed by lane of strep-tagged bTRPV3-pIRES2-AcGFP1 cells. A strong band appears at ~ 90 kDa after staining with strep-antibody. b) Fluorescence microscopy image shows HEK-293 cells reseeded at a lower concentration for patch clamp measurements after 48 hours of incubation with bTRPV3-pIRES2-AcGFP vector. Green fluorescence indicates a successful expression of the bTRPV3-pIRES2-AcGFP vector. c) Immunoblot of HEK-293 cell lysates 24 hours after transfection with bTRPV3-pcDNA5/TO vector.

HEK-293 cells were cultivated in Dulbecco’s modified Eagle’s medium supplemented with 10% (vol/vol) fetal bovine serum, 4 mmol∙l^-1^ glutamine and 100 units∙ml^-1^ of both penicillin and streptomycin (Biochrom, Berlin, Germany). Expression in both vector systems (pIRES2-AcGFP1 and pcDNA5/TO) was confirmed by immunoblotting (Fig 1A and 1C). HEK-293 cells were transiently transfected using poly ethylene imine (PEI) either with the vectors harbouring the bTRPV3 construct or the respective empty vectors and grown for 24 hours. Cells were subsequently harvested, washed twice in ice-cold phosphate-buffered saline (PBS) and resuspended in lysis buffer (50 mM Tris HCl pH 8.0, 150 mM NaCl, 1.2% Triton X-100, 0.1% SDS, 1 mM EDTA). Lysis was performed for 30 min at 4°C with gentle agitation followed by a clarifying spin (20 min, 14 000 rpm, 4°C). Proteins were resolved on 10% polyacrylamide-gels (SDS-PAGE) and electroblotted onto polyvinylidene difluoride (PVDF) membranes. A primary mouse antibody directed against the Strep-tag (1:2500, Qiagen, Hilden, Germany) in combination with a horseradish peroxidase (HRP)-conjugated secondary antibody (anti-mouse, 1:2000; from Cell Signaling Technology, Frankfurt, Germany) were used to detect bTRPV3. Proteins were visualized by use of the Clarity Western ECL Substrate (Bio-Rad, Munich, Germany).

For patch-clamping, subcloning was performed into a bicistronic vector allowing the simultaneous expression of green fluorescent protein (pIRES2-AcGFP1). Since successfully transfected cells emit a green fluorescent signal, they could be selectively targeted for patch-clamping (Fig 1B). For controls, the empty pIRES2-AcGFP1 vector was used without the inserted bTRPV3 gene. For each experimental day, HEK-293 cells from the same passage were transiently transfected with either bTRPV3 or the empty control vector using PEI and studied in parallel.

For calcium imaging, a tetracycline-regulated expression system was used for the transfection of HEK-293 cells (pcDNA 5/TO). Otherwise, the transfection procedure was as described above. The best protein expression efficiency for the pcDNA 5/TO-vector was reached 24 h post transfection (Fig 1C).

### Patch-clamp measurements

Patch-clamp experiments were performed essentially as previously described [30, 31] in a continuously perfused bath chamber allowing artefact-free solution changes. Cells were monitored with an inverted fluorescence microscope (Zeiss Axiovert). Borosilicate glass pipettes were pulled to a resistance of 3–5 MOhm using DMZ Universal Puller (Zeitz Instruments, Munich, Germany). Currents were recorded by an EPC 9 patch-clamp amplifier (HEKA Electronic, Lambrecht, Germany).

**Fig 2:**
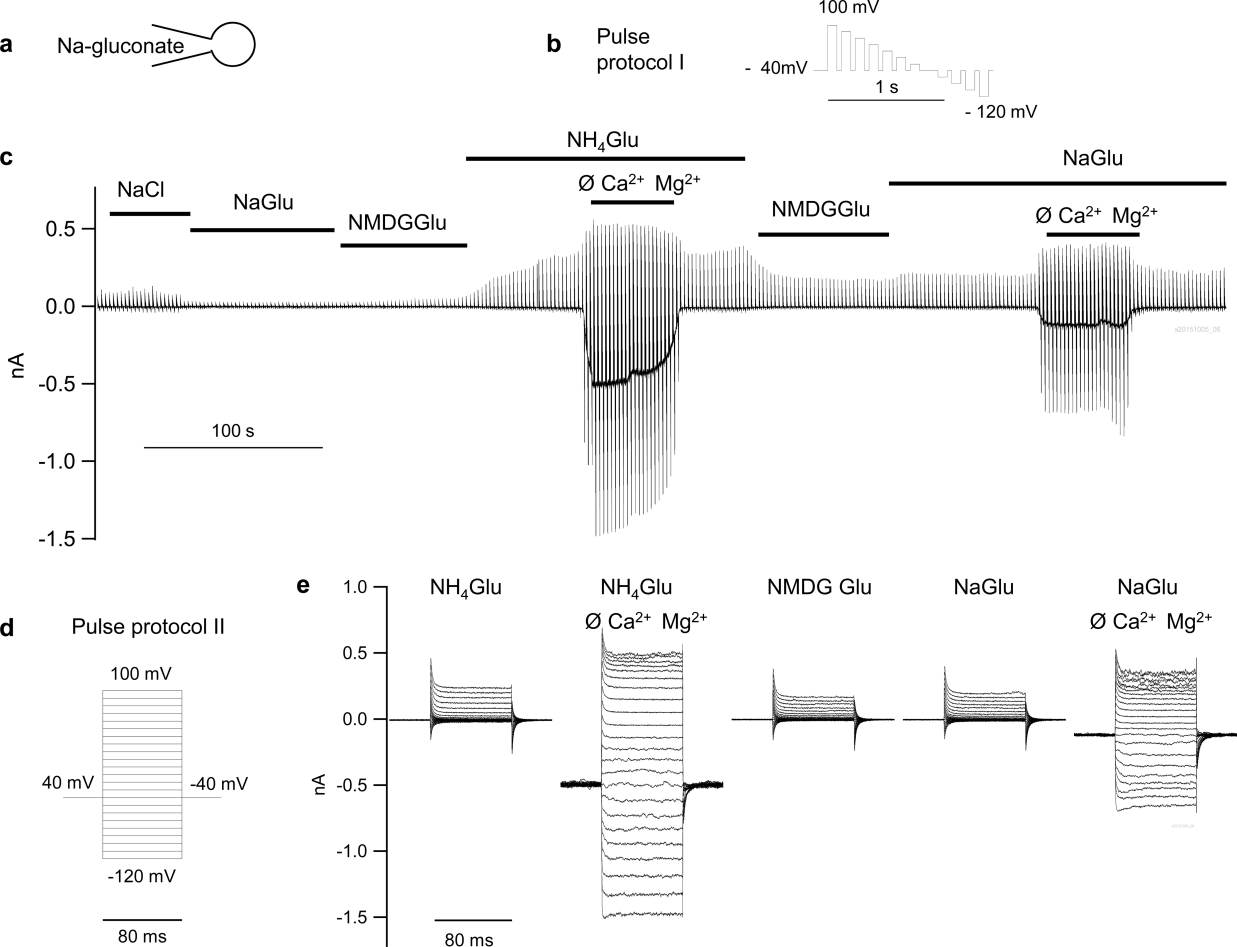
Whole cell measurements of HEK-293 cells expressing bTRPV3. Cells were filled with a Na-gluconate pipette solution (a) and superfused with various bath solutions as indicated. Cells were stimulated via consecutive application of the continuous pulse protocol I (b), subsequently merged. This yielded traces such as that in (c), which represents the original recording of one individual bTRPV3 cell. At regular intervals (66 s), pulse protocol I was briefly interrupted to run the high resolution classical step protocol II shown in (d), yielding current responses such as that shown in (e).

Pulse generation, data collection, filtering with a 2.9 kHz Bessel filter, and the correction for capacity and series resistance was automatically performed by Patchmaster Software (HEKA Electronic). For whole cell measurements, the protocol editor of Patchmaster was programmed to automatically switch every 66 seconds from a continuous protocol for assessing solution changes at low sampling rate (100 Hz) (protocol I, Fig 2B) to a classical pulse protocol for assessing channel kinetics at high sampling rate (5 kHz) with automatic compensation for capacitance and series resistance (protocol II, Fig 2D). Files from protocol I were subsequently merged to yield traces like that in Fig 2C. All patch-clamp experiments were performed at 23°C. After each cell overexpressing bTRPV3, a control cell was measured. For single-channel measurements, data were sampled at 10 kHz (protocol III).

#### Solutions

The conductance for NH_4_^+^ and the interaction between monovalent and divalent cations was tested using a Na-gluconate (NaGlu) pipette solution that consisted (in mmol∙l^−1^): 122 NaGlu, 15 NaCl, 1 NaH_2_PO_4_, 5 KCl, 10 HEPES, 0.9 MgCl_2_, 5 EGTA. In a second pipette solution designated as NMDG-gluconate or “NMDGGlu”, all but 1 mmol∙l^−1^ Na^+^ was replaced by NMDG^+^. The standard extracellular solution “NaCl” contained (in mmol∙l^−1^): 137 NaCl, 5 KCl, 1 NaH_2_PO_4_, 10 HEPES, 1.7 CaCl_2_, 0.9 MgCl_2_. Na^+^ was replaced by NMDG^+^ or NH_4_^+^ in the solutions designated as “NMDGCl” or “NH_4_Cl”. In gluconate based solutions, the Cl^−^ concentration was reduced from 142 to 21.8 mmol∙l^−1^ by replacement with gluconate^−^ using the commercial salt or via titration. Where indicated, 5 EGTA was used to replace Ca^2+^ in the continued presence of 0.9 mmol∙l^−1^ Mg^2+^. The Cl^−^ concentration was adjusted using NMDGCl. In high Ca^2+^ solutions, NaH_2_PO_4_ was omitted. The osmolality of all solutions was adjusted to 300 mosmol∙kg^−1^ using mannitol, the pH to 7.4 using Tris or HCl.

To investigate the action of agonists on bTRPV3, solutions from Macpherson et al. [32] were used (in mmol∙l^−1^): pipette: 140 CsCl, 10 HEPES, 1 MgATP, 5 EGTA, pH 7.4; bath solution: 140 NaCl, 5 KCl, 2 MgCl_2_, 10 HEPES, 5 EGTA, pH 7.4. To assess the impact of menthol and 2-APB on the conductance for K^+^ and NH_4_^+^, this recipe was varied in a few experiments by replacing CsCl with K-gluconate in the pipette and NaCl with NH_4_Cl in the bath.

Menthol, thymol, and carvacrol were freshly dissolved in ethanol on each experimental day and kept on ice until immediately prior to the experiment, when the stock solution was added to the bath solution in the perfusion system at 1:1000. 2-Aminoethoxydiphenyl borate (2-APB) was dissolved in methanol.

Solutions for single-channel patch-clamp measurements were composed according to Doerner et al. [33]. The “NaCl” pipette solution contained (in mmol∙l^−1^): 145 NaCl, 5 CsCl, 1 EGTA, 20 HEPES. NaCl was replaced by NH_4_Cl in the solution designated as “NH_4_Cl”. In a further solution (“NH_4_Glu”) 130 mmol∙l^−1^ of Cl^−^ were replaced by gluconate^−^. Both solutions were also used in the bath, where indicated by these abbreviations. Additionally, the following solutions were used: 1.) “NMDGGlu”: 5 CsCl, 1 EGTA, 20 HEPES, 15 NaCl, 130 NMDGGlu; 2.) “CaCl_2_”: 5 CsCl, 1 EGTA, 20 HEPES, 75.2 CaCl_2_; 3.) “MgCl_2_”: 5 CsCl, 1 EGTA, 20 HEPES, 75.2 MgCl_2_; 4.)“NH4Glu 1.5 Ca^2+^”: 5 CsCl, 1 EGTA, 20 HEPES, 1.5 CaCl_2_, 12 NH_4_Cl, 131 NH_4_Glu, 5.) “NaCl 1.5 Mg^2+^”: 5 CsCl, 20 HEPES, 1.5 MgCl_2_, 145 NaCl, 6.) “NaCl 1.5 Ca^2+^”: 5 CsCl, 20 HEPES, 1.5 CaCl_2_, 145 NaCl.

#### Data analysis

Data processing was performed using Igor Pro 6.2.2.2 (WaveMetrics Inc., Lake Oswego, USA) and Sigma Plot for Windows 11.0 (Systat Software 11.0, Erkrath, Germany). All potentials were corrected for liquid junction potential [34].

*Whole cell measurements*: Where not indicated otherwise, the term “outward current” is used to designate the current density at +100 mV. This reflects either cations (e.g. Na^+^) flowing out of the pipette into the bath or anions (e.g. Cl^−^) flowing out of the bath into the pipette or the sum of both. Likewise, current densities designated as “inward currents” were measured at −100 mV and reflect the reverse flow of anions and cations. To obtain current density, absolute current values were divided by the capacity of each cell. Alternately, currents were normalized to the initial current at +100 mV in NaCl solution, which was set to 100%. Continuous pulse files (protocol I) were merged using Igor to yield an overview of the response of the cell to changes in solution. For all other purposes, data were obtained from classical pulse files (protocol II) via Patchmaster software, set to export the mean of the current response in the latter third of each voltage step (from 60 ms to 72 ms). Reversal potentials were calculated by linear interpolation between the points immediately above and below a current of zero in the corresponding IV-curve. Measurements with a series resistance of under 4 or over 15 MOhm were excluded as well as cells in which inward current in NMDG-gluconate solution was more negative than −75 pA/pF (measured at −120 mV) suggesting a leaky seal. In addition, one bTRPV3 and one control cell were removed since the reversal potential in symmetrical Na-gluconate was < −35 mV, indicating an error with offset correction. Assuming that the cations do not interact with each other in the channel pore, the relative permeability ratios for monovalent ions and Ca^2+^ were calculated according to standard methods from the reversal potentials using Goldman-Hodgkin-Katz theory [28, 35].

*Single-channel measurements*: An Igor macro was used to fit amplitude histograms to a Gaussian distribution with the distance of the maxima giving the unitary current of one channel opening. These values were cross-checked by directly measuring the size of single-channel openings in the original traces. The unitary currents were plotted against the pipette potentials in IV-curves. For symmetrical solutions, the slope of the linear regression equals the conductance. For asymmetrical solutions, a Goldmann-Hodgkin-Katz (GHK) fit was used as follows:

Let P_A_ and P_B_ designate the permeabilities of two cations A and B with activity coefficients α_A_ and α_B_ and valencies z_A_ and z_B_, while [cA]_i_ and [cB]_i_ are the concentrations in the pipette, and [cA]_o_ and [cB]_o_ the concentrations in the bath. Further, let [A]_i_ = z_A_∙ α_A_∙[cA]_i_, [A]_o_ = z_A_∙ α_A_∙[cA]_o_, [B]_i_ = z_B_∙ α_B_∙[cB]_i_ and [B]_o_ = z_B_∙ α_B_∙[cB]_o_.

Again assuming no interaction between the cations, the resulting current is given by:

$$I=\frac{E_{p}∙F^{2}}{R∙T}∙\left(\frac{P_{A}∙{\left[A\right]}_{i}+P_{B}∙{\left[B\right]}_{i}-(P_{A}∙{\left[A\right]}_{o}+(P_{B}∙{\left[B\right]}_{o})∙\exp\left(-E_{p}∙\frac{F}{R∙T}\right)}{1-\exp\left(-E_{p}∙\frac{F}{R∙T}\right)}\right)$$ (1)

Here, E_p_ designates the pipette potential (corrected for liquid junction potential), F the Faraday constant, and T the absolute temperature.

In symmetrical solution with just one ion ([X]_i_ = [X]_o_, no other ions), this equation can be simplified to:

$$I=\frac{E_{p}∙F^{2}}{R∙T}∙P_{X}∙\left[X\right]$$ (2)

The values for P resulting from the fit of currents in asymmetrical solutions were used to predict the conductance C in symmetrical 145 mmol∙l^−1^ solution at a given concentration of the ion ([X]):

$$C_{X}=\frac{I}{E_{p}}=\frac{F^{2}}{R∙T}∙P_{X}∙\left[X\right]$$ (3)

Open probability (NPo) was evaluated for symmetrical NH_4_Cl and NaCl solutions by measuring the area under the current trace and dividing it by the single-channel conductance for the corresponding pipette potential. For comparisons, data were normalized to the value of NPo at the beginning of the series.

### Fura-2-assisted intracellular Ca^2+^ measurements

HEK-293 cells were transiently transfected with an empty vector control or the pcDNA5TO-bTRPV3 vector using PEI for 24 hours as outlined above. As before, after each measurement with overexpressing cells, control cells were measured. Cells were loaded in HBSS (Hanks balanced salt solution) with 5 μmol∙l^-1^ of the ratiometric, membrane-permeable calcium indicator FURA-2 AM (Thermo Fischer Scientific, Bremen, Germany) at 37°C for 20 minutes. Afterwards cells were washed and incubated in HBSS for further 30 minutes at 37°C to allow complete de-esterification of the dye. Loaded cells were centrifuged (3 min, 300 g) and resuspended in Ca^2+^- and Mg^2+^- free HBSS. Measurements were performed at 37°C in 3-ml cuvettes containing 2 ml cell suspension under stirring. The 340/380 nm ratios were acquired with a spectrofluorometer LS55 (PerkinElmer, Rodgau, Germany) equipped with a fast-filter accessory at a sampling rate of 6.25 Hz.

After 300 seconds, 1 mmol∙l^-1^ menthol was added to the cuvette. For calibration, 50 μmol∙l^-1^ digitonin was used to lyse the cells after which 1 mmol∙l^-1^ Ca^2+^ was added to saturate all available Fura-2 with Ca^2+^, thereby yielding the maximum value. The minimum value was determined after addition of 50 mmol∙l^-1^ Triton X-100 with subsequent addition of 50 mmol∙l^-1^ EDTA to chelate all calcium in the suspension. Ca^2+^ concentrations were calculated using the FL WinLab software provided by the LS55 based on the method by Grynkiewicz [36] and using a specific Kd value of 224 nM. After filtering the data using a 250 point median filter, each curve was corrected for drift by subtracting the slope of the baseline before application of menthol from all data (IgorPro).

#### Statistical analysis

Data were tested for normality using Kolmogorov–Smirnov test (SigmaPlot 11.0). Comparisons between two groups were performed using unpaired Student’s t-tests if normally distributed, otherwise the Wilcoxon Signed Rank Test or Mann-Whitney Rank Sum Test was used. In cases where different solutions were consecutively applied, normally distributed data were evaluated using one-way repeated measures ANOVA and the Tukey Test for multiple pairwise comparisons. Not normally distributed paired data were tested using ANOVA on Ranks followed by the Student-Newman-Keuls method for multiple comparisons.

## Results

### Whole cell experiments

#### Conductance to Na^+^ and NH_4_^+^

**Fig 3:**
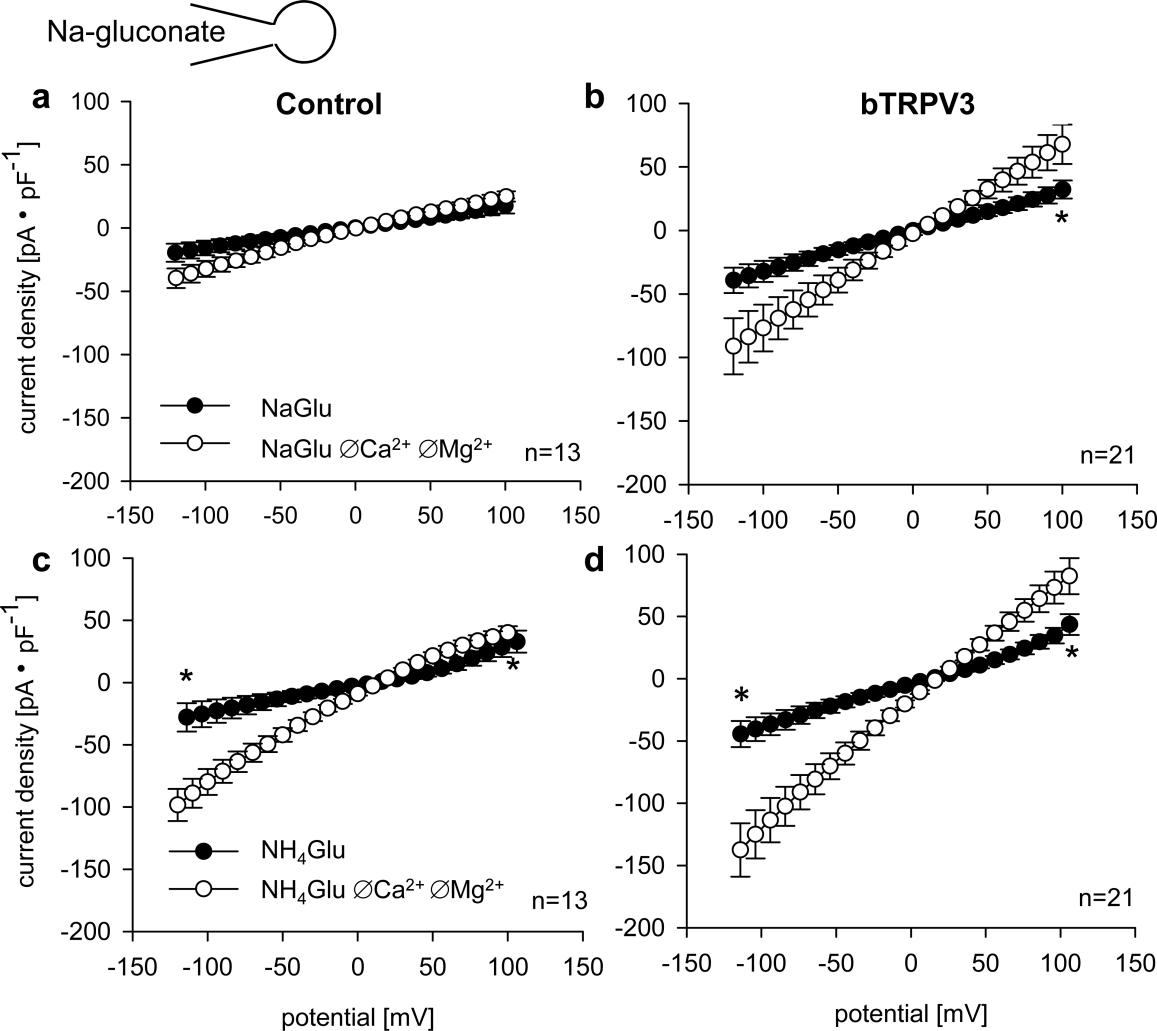
Current-voltage plots of whole cell measurements from Fig 2. The figures give the mean current values (± SEM) of control cells (a, c) and cells expressing bTRPV3 (b, d) in Na-gluconate (a, b) or NH_4_-gluconate (c, d) bath solutions with (●) and without (○) divalent cations, as indicated. (*: p < 0.05, tested for −100 mV and +100 mV).

In a first series of whole cell experiments, the conductance to Na^+^ and NH_4_^+^ was studied in HEK-293 cells transfected either with bTRPV3 or the control vector. Cells were filled with a sodium gluconate (NaGlu) pipette solution and gluconate was used to substitute for chloride in all bath solutions except for the initial standard NaCl solution. Results of a representative measurement are shown in Fig 2, the mean IV-curves in Fig 3, current densities in Table 1, and reversal potentials in Table 2. Table 2 also gives the rectification index, which was calculated by dividing the current density at +100 mV by the current density at −100 mV. Throughout, there was a trend for higher current density in the bTRPV3 cells that reached significance level when divalent cations were removed from the solution.

**Table 1:** Outward currents and inward current densities in HEK-293 cells expressing bTRPV3 (n = 21) or the control vector (n = 13) (pipette solution: Na-gluconate).

Pipette: NaGlu	outward current (+ 100 mV) pA∙pF^-1^			inward current (- 100 mV) pA∙pF^-1^		
bath:	bTRPV3	control	p	bTRPV3	control	p
NaCl	40.7 ± 8.8^a^	24.9 ± 6.8^a^	0.34	-36.5 ± 9.1^a^	-18.9 ± 6.0^ad^	0.32
NaGlu	32.2 ± 7.1^b^	17.4 ± 5.9^b^	0.18	-32.3 ± 8.3^b^	-15.8 ± 5.8^b^	0.40
NMDGGlu	27.2 ± 5.8^c^	16.6 ± 5.4^b^	0.46	-17.2 ± 4.6^c^	-9.2 ± 3.8^c^	0.46
NH_4_Glu	43.6 ± 8.4^a^	32.8 ± 8.9^a^	0.48	-36.5 ± 8.7^a^	-22.9 ± 9.4^d^	0.62
NH_4_Glu ∅ Ca^2+^∅Mg^2+^	82.4 ± 14.5^d^	40.0 ± 5.2^c^	0.04	-113.6 ± 17.8^d^	-79.8 ± 10.4^e^	0.24
NH_4_Glu (2)	69.3 ± 11.9^e^	34.5 ± 4.3^d^	0.06	-73.2 ± 16.4^e^	-20.6 ± 3.3^f^	0.16
NMDGGlu (2)	43.2 ± 7.4^f^	20.8 ± 2.9^a^	0.06	-31.4 ± 8.3^f^	-10.1 ± 2.1^c^	0.98
NaGlu (2)	52.0 ± 10.4^f^	20.7 ± 3.0^a^	0.07	-50.9 ± 13.6^b^	-14.3 ± 3.0^b^	0.15
NaGlu ∅ Ca^2+^∅Mg^2+^	67.9 ± 15.6^e^	25.0 ± 4.0^a^	0.02	-76.8 ± 18.5^e^	-32.3 ± 6.3^f^	0.06
NaGlu (3)	56.4 ± 14.9^a^	21.1 ± 3.2^a^	0.07	-54.6 ± 17.7^a^	-16.6 ± 3.3^ab^	0.20
NaCl (2)	61.8 ± 18.3^a^	24.1 ± 3.9^a^	0.31	-58.3 ± 20.5^a^	-19.5 ± 4.1^ab^	0.33

Values are given as means ± SEM.

Different superscripts refer to significant differences within one column (p < 0.05).

P values are given for control versus bTRPV3.

The numbers in parentheses reflect repeated application of the same bath solution.

**Table 2:** Reversal potentials and rectification index of cells expressing bTRPV3 (n = 21) or the control vector (n = 13) (pipette solution: Na-gluconate).

Pipette: NaGlu	Reversal Potential mV			Rectification Index I(100mV)/I(-100mV)		
Bath:	bTRPV3	control	p	bTRPV3	control	p
NaCl	-15.0 ± 2.6^a^	-11.0 ± 0.6^a^	0.57	-2.0 ± 0.4^a^	-1.9 ± 0.4^a^	0.67
NaGlu	-2.0 ± 1.8^b^	-1.3 ± 1.6^b^	0.57	-2.2 ± 0.5^a^	-1.7 ± 0.4^b^	0.46
NMDG Glu	-23.0 ± 4.0^c^	-22.0 ± 4.4^c^	0.62	-6.6 ± 2.8^b^	-4.2 ± 1.5^c^	0.92
NH4Glu	13.5 ± 2.0^d^	15.1 ± 2.4^d^	0.99	-3.2 ± 0.9^a^	-2.2 ± 0.5^a^	0.62
NH4Glu ∅ Ca^2+^∅Mg^2+^	17.6 ± 1.0^e^	20.4 ± 2.0^e^	0.13	-1.0 ± 0.3^c^	-0.5 ± 0.1^d^	0.01
NH_4_Glu (2)	17.2 ± 1.0^e^	18.1 ± 2.4^e^	0.80	-2.8 ± 0.8^a^	-2.1 ± 0.4^e^	0.07
NMDGGlu (2)	-15.3 ± 2.8^a^	-14.5 ± 2.8^a^	0.41	-4.8 ± 1.3^b^	-2.9 ± 0.5^f^	0.34
NaGlu (2)	0.8 ± 1.7^f^	2.5 ± 2.1 ^f^	0.75	-3.1 ± 0.9^d^	-2.0 ± 0.4^a^	0.13
NaGlu ∅Ca^2+^∅ Mg^2+^	1.2 ± 1.7^f^	1.1 ± 1.7^f^	0.74	-1.2 ± 0.2^e^	-1.1 ± 0.3^g^	0.17
NaGlu (3)	3.1 ± 1.1^f^	3.0 ± 2.2^f^	0.67	-2.9 ± 1.0^a^	-1.8 ± 0.3^a^	0.89
NaCl (2)	-7.4 ± 1.4^g^	-7.3 ± 2.0^g^	0.85	-2.2 ± 0.5^a^	-1.7 ± 0.3^a^	0.93

Values are given as means ± SEM.

Different superscripts refer to significant differences within one column (p < 0.05).

P values are given for control versus bTRPV3.

The numbers in parentheses reflect repeated application of the same bath solution.

In both types of cells, a switch from Cl^−^ to gluconate^−^ resulted in a significant reduction in outward current with concomitant depolarisation of the reversal potential, reflecting a conductance to Cl^−^. Interestingly, a switch from Na^+^ to NMDG^+^ induced a significant decrease in outward current in bTRPV3 cells but not in controls, possibly reflecting a certain negative interaction between the two ions in the channel pore of bTRPV3. As calculated from the shift in reversal potential [35], relative permeabilities were p(Na^+^)/p(NMDG^+^) = 2.7 ± 0.4 for bTRPV3 with the value for control cells at 2.9 ± 0.5 (p > 0.1). Notably, this value is calculated at the reversal potential, where current through the channel is negligible so that the effects of a negative interaction of the two ions cannot be assessed. Application of NH_4_^+^ induced a significant rise in inward current with a concomitant depolarisation in both cell types, reflecting an influx of the NH_4_^+^ ion. Outward current also rose, which may reflect effects of changes in pH or volume on Na^+^ conducting channels, or stimulatory effects of an increase in the concentration of permeant ions. Relative permeabilities were p(NH_4_^+^)/p(NMDG^+^) = 8.8 ± 3.4 and 10.0 ± 4.7, significantly higher than p(Na^+^)/p(NMDG^+^) in both groups (p < 0.001 and p = 0.007), but not different from each other (p > 0.1 versus bTRPV3).

After removal of divalent cations, inward and outward currents rose significantly in both types of cells with effects significantly higher in cells expressing bTRPV3 (Table 1 and Fig 3).

Conversely, the reversal potential and thus the relative permeability ratio did not change. This reflects the fact that efflux of Na^+^ from the cells rose in proportion to the influx of NH_4_^+^ so that the overall membrane potential did not change. However, the rectification index showed significant differences and approached unity in cells expressing bTRPV3 in line with the non-selective nature of the conductance. Conversely, control cells expressed a significantly lower relative conductance to Na^+^, leading to inward rectification. Both cell types had the highest rectification index in the bath solutions containing Ca^2+^, suggesting a voltage-dependent interaction of Ca^2+^ with the conductance for monovalents (Table 2).

In conjunction, both bTRPV3 expressing cells and controls expressed a considerable conductance to NH_4_^+^, suggesting a participation of endogenous channels to total conductance [37]. However, the membrane conductance of cells expressing bTRPV3 showed a greater response to the removal of divalent cations, reflecting a higher contribution of the non-selective bTRPV3 channel.

#### Effects of various agonists on bTRPV3

**Fig 4:**
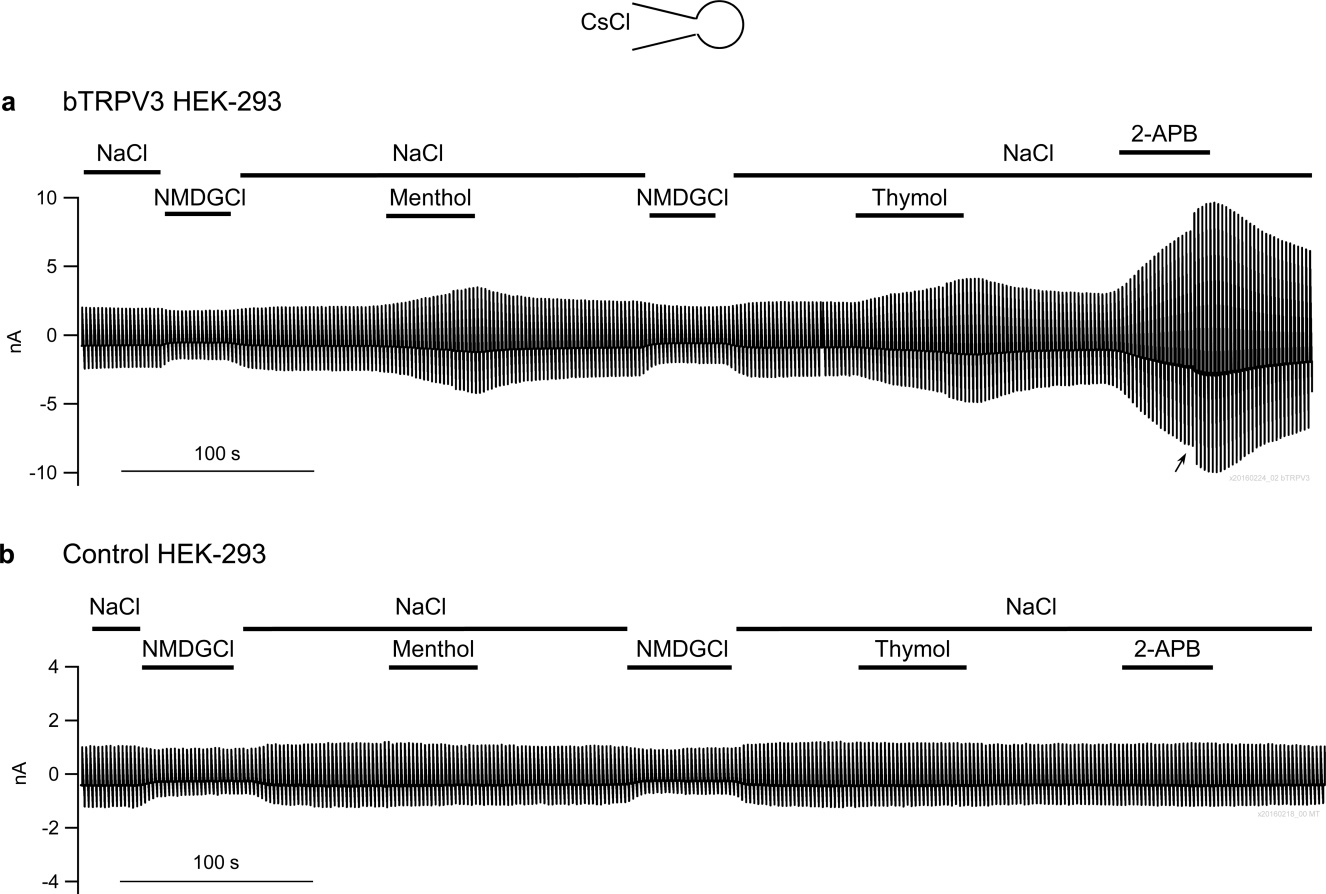
Response to various agonists of bTRPV3. Cells were filled with a CsCl pipette solution and stimulated with the continuous pulse protocol I. a) Original recording of a cell expressing bTRPV3, showing the response to application of menthol, thymol (both 1 mmol∙l^−1^), and 2-APB (0.3 mmol∙l^−1^) in NaCl bath solution. In response to 2-APB, the current rose dramatically, requiring an interruption of the measurement to readjust the gain (arrow). b) Original recording of a control cell transfected with the empty vector.

The human homologue of TRPV3 can be stimulated by a variety of agonists that include menthol, thymol, 2-APB, and carvacrol [32, 38]. Cells expressing bTRPV3 and controls were filled with a CsCl solution and superfused with a Ca^2+^-free NaCl bath solution, all as reported by Macpherson et al. [32]. In a first series, menthol (1 mmol∙l^−1^), thymol (1 mmol∙l^−1^), and 2-APB (300 μmol∙l^−1^) were added consecutively via continuous perfusion with washout steps in between (Fig 4). Cells expressing bTRPV3 (n = 16) showed an inward current at −100 mV of −144 ± 54 pA∙pF^−1^, an outward current at +100 mV of 161 ± 57 pA∙pF^−1^, and a reversal potential of −7.6 ± 0.7 mV, reflecting that permeability to Cs^+^ (p(Cs^+^)) was greater than that to Na^+^(p(Na^+^)) (Table 3). Values measured in control cells (n = 11) were similar (−141 ± 24 pA∙pF^−1^, 157 ± 27 pA∙pF^−1^, −7.3 ± 1.4 mV, all p > 0.1 versus bTRPV3). In both cell types, replacement of Na^+^ by NMDG^+^ resulted in a significant hyperpolarisation of reversal potential to similar values (−21.0 ± 1.5 and −19.1 ± 3.3 mV for bTRPV3 and control) with the permeability ratio p(Na^+^)/p(NMDG^+^) at 1.7 ± 0.1 and 1.6 ± 0.1, respectively.

**Table 3:** Effects of TRPV3 agonists on whole cell currents of HEK-293 cells expressing bTRPV3 or the control vector (pipette solution: CsCl).

Pipette: CsCl	current at + 100 mV % of initial current		Cont. vs bTRPV3	current at—100 mV % of initial current		Cont. vs bTRPV3	number of cells n	
bath:	bTRPV3	control	P value	bTRPV3	control	P value	bTRPV3	control
NaCl (1)	100 ± 0^a^	100 ± 0^a^		-86 ± 3^a^	-88 ± 3^a^	0.604	16	11
Menthol	195 ± 22^b^	103 ± 5^a^	0.001	-150 ± 20^b^	-88 ± 4^a^	0.008	16	11
NaCl (2)	146 ± 16^c^	105 ± 7^a^	0.087	-119 ± 12^b^	-89 ± 6^a^	0.087	15	11
NMDG Cl	130 ± 11^a^	97 ± 8^a^	0.057	-74 ± 8^c^	-65 ± 7^b^	0.537	16	10
NaCl (3)	147 ± 13^d^	102 ± 1^a^	0.022	-121 ± 10^ab^	-126 ± 4^a^	0.098	16	9
Thymol	251 ± 34^e^	106 ± 1^a^	0.001	-188 ± 23^d^	-143 ± 5^a^	0.020	13	9
NaCl (4)	161 ± 21^d^	107 ± 1^a^	0.082	-128 ± 16^b^	-141 ± 5^a^	0.249	12	8
2-APB	657 ± 11^f^	96 ± 1^a^	0.002	-521 ± 82^e^	-81 ± 1^a^	0.001	8	7
NaCl (5)	642 ± 37^d^	95 ± 1^a^	0.002	-550 ± 33^e^	-81 ± 1^a^	0.009	8	7
NaCl (1)	100 ± 0^a^	100 ± 0^a^		-81 ± 6^a^	-74 ± 10^a^	0.730	5	4
Carvacrol	284 ± 30^b^	104 ± 6^a^	0.016	-210 ± 31^b^	-77 ±7^a^	0.016	5	4
NaCl (2)	159 ± 15^c^	102 ± 7^a^	0.016	-118 ± 19^c^	-75 ±7^a^	0.190	5	4

Values are given as means ± SEM.

Different superscripts refer to significant differences within one column (p < 0.05).

P values are given for control versus bTRPV3.

The numbers in parentheses reflect repeated application of the same bath solution.

Pronounced differences between the two types of cells emerged during stimulation with the agonists. In cells expressing bTRPV3, the addition of menthol had a significant effect on inward current (−249 ± 59 pA∙pF^−1^, p < 0.001) and outward current (289 ± 62 pA∙pF^−1^, p < 0.001) with partial washout after return to NaCl (Fig 4A). In contrast, control cells were not affected by the addition of menthol (−143 ± 27 pA∙pF^−1^, 168 ± 33 pA∙pF^−1^, p > 0.1) (Fig 4B). After application of menthol, the reversal potential of bTRPV3 cells was significantly lower (−8.7 ± 0.9 mV) than in controls (−7.3 ± 1.4 mV, p = 0.03). Since Cl^−^ concentrations were identical in the solutions used, this argues for a higher conductance of bTRV3 cells to the Cs^+^ in the pipette solution. Similarly, thymol and 2-APB significantly stimulated currents in bTRPV3 expressing cells, but not in controls, with differences in current densities passing testing for significance after application of 2-APB (bTRPV3: −292 ± 46 pA∙pF^−1^ and 356 ± 56 pA∙pF^−1^ versus control: −97 ± 23 pA∙pF^−1^ and 112 ± 27 pA∙pF^−1^, p < 0.005). After normalization to the initial current in NaCl solution at +100 mV, all differences between the response of overexpressing cells and controls tested for significance (Table 3). Likewise, carvacrol (1 mmol∙l^−1^) significantly enhanced inward and outward current in bTRPV3 cells, but not in controls (Table 3). In conjunction, while control and bTRPV3 cells had similar overall conductance levels, only the conductance of cells expressing bTRPV3 could be stimulated by menthol, thymol, carvacrol, or 2-APB.

Subsequently, further six cells expressing bTRPV3 were investigated using a more physiological K-gluconate pipette solution. Replacement of Na^+^ in the bath by NH_4_^+^ resulted in an increase in inward current at −100 mV from −70.3 ± 30.4 pA∙pF^−1^ to −97.9 ± 34.9 pA∙pF^−1^, while reversal potential depolarised from −17.5 ± 3.5 mV to −6.3 ± 3.1 mV, both reflecting influx of NH_4_^+^ (all p < 0.05). Outward current at +100 mV rose from 78.6 ± 30.8 pA∙pF^−1^ to 98.9 ± 29.4 pA∙pF^−1^ (p < 0.05). Application of menthol (1 mmol∙l^−1^) resulted in a further increase in inward and outward currents to −130.7 ± 36.8 pA∙pF^−1^ and +136.2 ± 32.5 pA∙pF^−1^, respectively (p < 0.05), with values of −123.0 ± 35.2 pA∙pF^−1^ and 125.9 ± 29.9 pA∙pF^−1^ observed after washout of menthol with NH_4_Cl solution (p > 0.05). Reversal potential depolarised to −2.0 ± 2.7 mV, with return to −4.1 ± 2.5 mV after washout (both p < 0.05). Subsequent application of 2-APB (300 μmol∙l^−1^ in NH_4_Cl solution) led to increases in both inward current (−512.6 ± 144.0 pA∙pF^−1^), outward current (437.8 ± 112.5 pA∙pF^−1^), and reversal potential (−2.0 ± 2.3 mV) with return to values of −177.6 ± 39.6 pA∙pF^−1^, 189.0 ± 33.2 pA∙pF^−1^, and −6.4 ± 2.7 mV after washout (all p < 0.05). In four cells, menthol could be subsequently applied a second time, yielding similar results. In conjunction, these values suggest that HEK-293 cells expressing bTRPV3 conduct NH_4_^+^ with p(NH_4_^+^)/p(Na^+^) = 1.5 ± 0.1. Menthol or 2-APB reversibly stimulated both the influx of NH_4_^+^ and the efflux of K^+^ with p(NH_4_^+^)/p(Na^+^) rising significantly by a factor of about 2 (or 1.90 ± 0.22 and 1.88 ± 0.15, respectively).

#### NMDG-gluconate pipette solution

**Fig 5:**
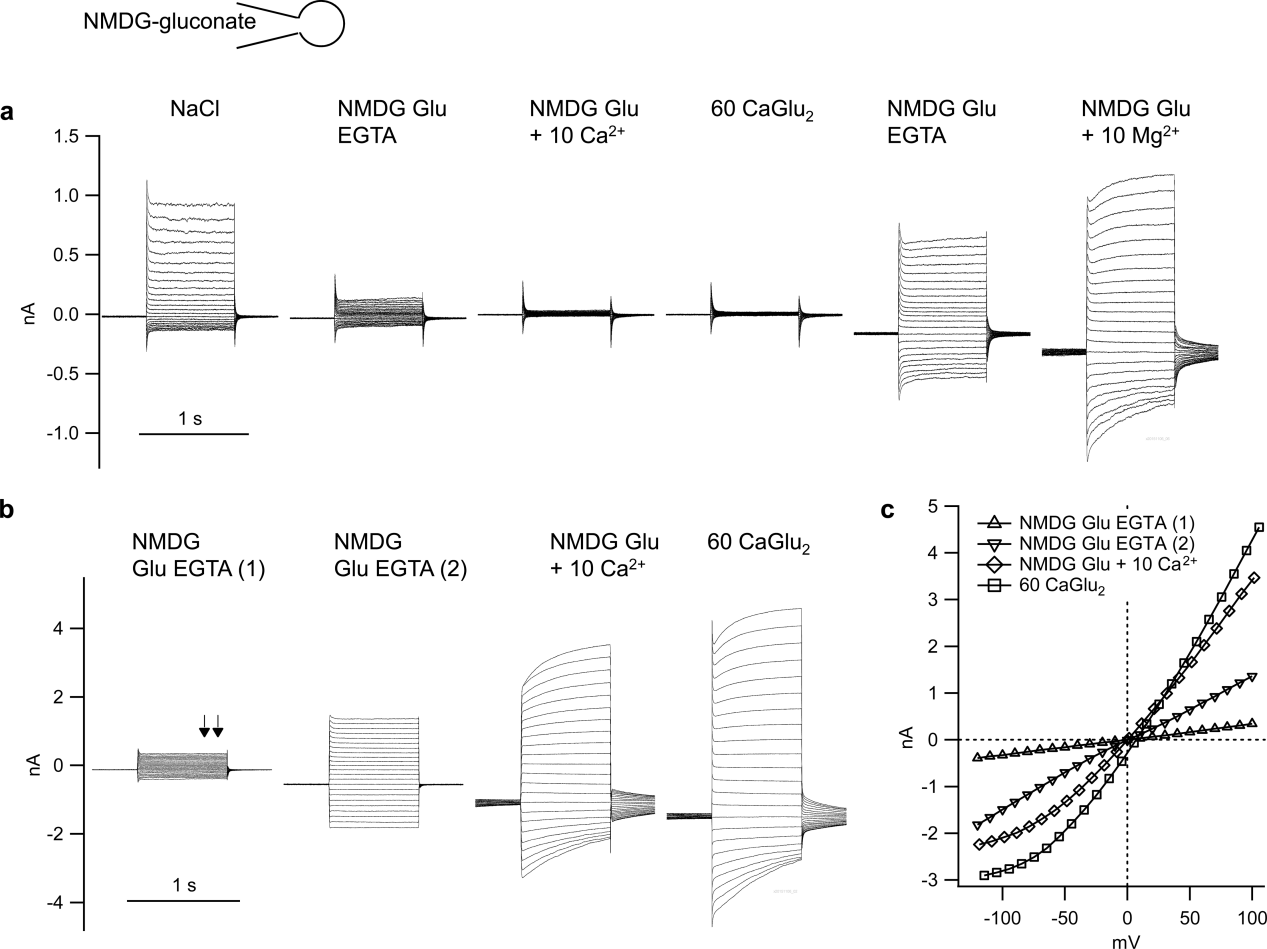
Original recordings of two bTRPV3 cells filled with NMDG-gluconate solution. a) As before, replacement of chloride by gluconate resulted in a decrease in outward current at positive potentials, suggesting expression of chloride channels. Both addition of Ca^2+^ (10 mmol∙l^−1^) and replacement by Ca-gluconate_2_ (60 mmol∙l^−1^) had a strong blocking effect, with washout to levels higher than before. The current could be partially blocked by Mg^2+^, arguing against a rupture of the seal. Note the current kinetics with amplitude decreasing after a hyperpolarising pulse suggesting that Mg^2+^ is being drawn into the channel pore by the negative membrane voltage. Conversely, a depolarising voltage pulse leads to a relief of the block. In both cases, the currents appear curved in contrast to the linear kinetics of currents in pure NMDG-gluconate solution. b) Second cell, showing a spontaneous activation of inward and outward current amplitude by a factor of about four between the first set of pulses in NMDG-gluconate EGTA solution (1) and a second set (2) that was run 100 seconds later. Current amplitude continued to increase both after addition of 10 mmol∙l^−1^ Ca^2+^ to the NMDG-gluconate solution, and after replacement of NMDG-gluconate by Ca-gluconate_2_. Both the current kinetics and the tail currents indicate a partial voltage-dependent block. The arrows above the first set of pulses indicate the interval used for the determination of mean current amplitude for the current-voltage plot, which is depicted in c), showing rectification as a further sign of a voltage-dependent block. The reversal potential in 60 mmol∙l^−1^ Ca-gluconate_2_ solution was 7.1 mV, revealing permeability to Ca^2+^.

To assess permeation for NMDG^+^ and Ca^2+^, the pipette solution was changed to NMDG-gluconate. In NaCl solution, the reversal potential was close to zero in both cell types, reflecting both influx of Na^+^ and influx of Cl^−^. In symmetrical NMDG-gluconate solution, current responses were highly variable and tended to increase over time (Fig 5) (p < 0.1). These currents were clearly not pure leak currents since addition of 10 mmol∙l^−1^ Ca^2+^ or Mg^2+^ had clear, albeit variable, blocking effects (p < 0.05) (Fig 5A and 5B). In some cases, the blocking effects were almost complete, albeit transient, as could be seen after application of Ca^2+^ in Fig 5A. Conversely, the subsequent application of Mg^2+^ in Fig 5A was not sufficient to block the general trend for an increase in current amplitude. Again, both the current kinetics and the appearance of tail currents argue against a simple leak and for a current mediated by either gluconate^−^ or NMDG^+^. Fig 5A clearly shows how in the presence of Mg^2+^, currents decreased after a hyperpolarising voltage step. Conversely, current amplitude increased with time after depolarisation suggesting a release of blocking Mg^2+^ ions from the pore of a channel permeable to NMDG^+^. In addition, tail currents were visible after a return to the original voltage level, all in line with a model in which the interaction of the blocking ion with the filter region is rapidly modulated by the membrane potential (see S1 Fig). Similar effects can be seen in Fig 5B, where the Ca^2+^ is the blocking ion.

Both cell types were depolarised significantly (p < 0.01) by adding Ca^2+^ to an NMDG-gluconate solution. In bTRPV3 cells (n = 11), reversal potential rose from 2.1 ± 2.3 mV (no Ca^2+^) to 8.4 ± 5.4 mV (10 mmol∙l^-1^ Ca^2+^) to 10.6 ± 3.0 (60 mmol∙l^-1^ Ca^2+^). In control cells (n = 5), effects of an addition of Ca^2+^ were significantly higher, with reversal potential rising from -0.1 ± 2.8 mV (no Ca^2+^) to 12.7 ± 4.5 mV (10 mmol∙l^-1^ Ca^2+^, p = 0.04 versus bTRPV3) to 25.0 ± 8.1 mV (p = 0.11 versus bTRPV3). This result reflects the poor selectivity of cells expressing the non-selective bTRPV3 channel.

As established in the field [28, 35] and using the shift in reversal potential after addition of 10 mmol∙l^−1^ Ca^2+^, we obtained relative permeability ratios of p(Ca^2+^)/p(NMDG^+^) = 1.7 ± 0.2 for bTRPV3 and 5.7 ± 2.6 for control cells (p = 0.036). Similar values (2.1 ± 0.4 and 5.5 ± 1.4) were calculated from the experiments with 60 mmol∙l^−1^ Ca^2+^. Using the data obtained for p(Na^+^)/p(NMDG^+^) above, it is possible to estimate p(Ca^2+^)/p(Na^+^), yielding ≈ 0.77 for bTRPV3 cells and ≈ 1.97 for controls. As will be discussed, these values must be treated with caution since Ca^2+^ interacted with NMDG^+^ so that the independence principle did not hold.

### Inside-out configuration

#### NH_4_Cl pipette solution

**Fig 6:**
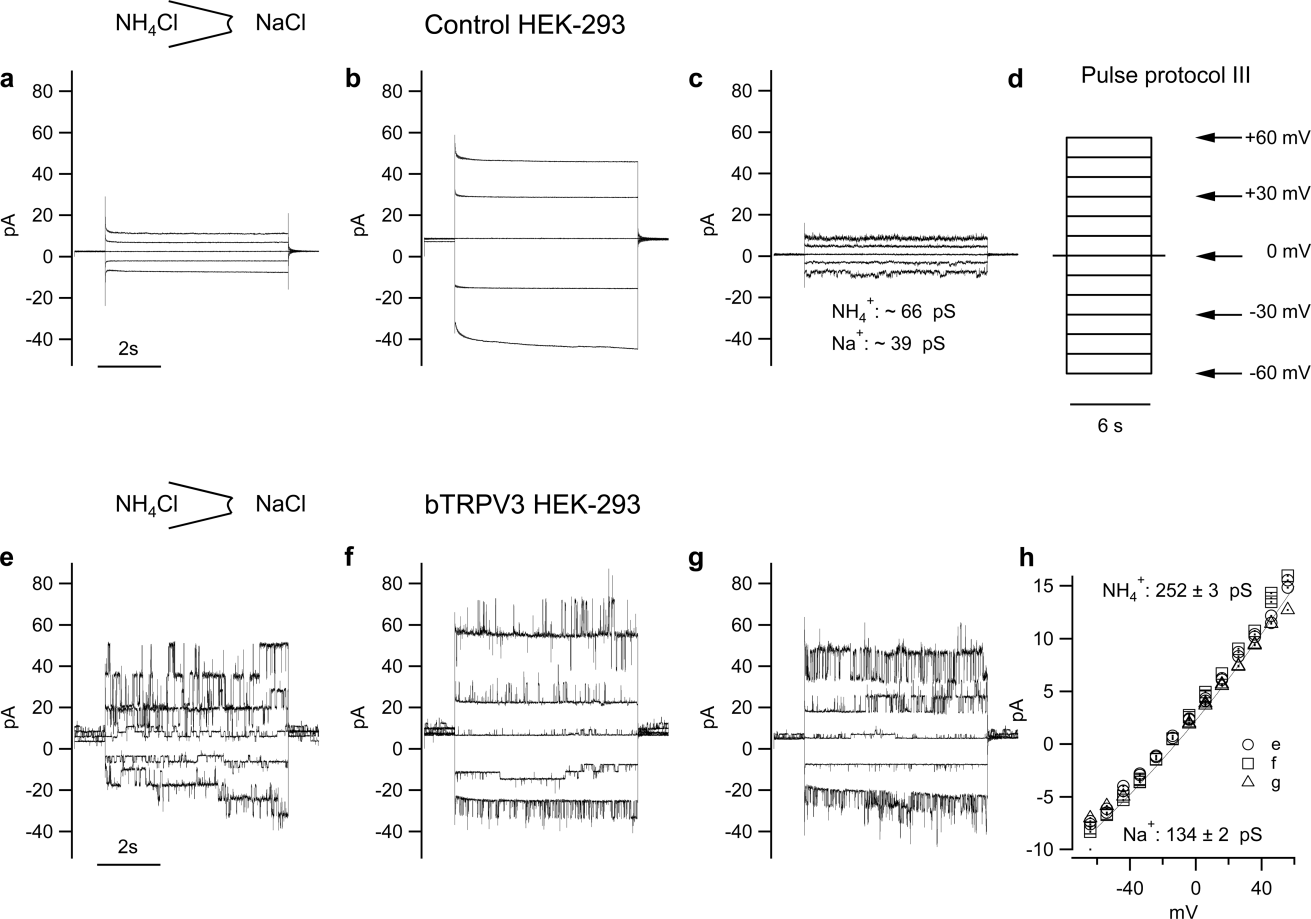
Inside-out patch-clamp measurements from control and bTRPV3 HEK-293 cells. All measurements were made with NH_4_Cl in the pipette and NaCl in the bath, and exposed to the pulse protocol III shown in d. For clarity, only the current responses to the pulses at –60, −30, 0, +30, and +60 mV are shown in the figures. a) patch from control cell, showing no clear sign of channel activity. b) patch from another control cell. Although no clear single-channel events can be seen, the kinetics of the currents suggest activity of very small channels. c) patch from a third control cell. Multiple peaks emerged in the amplitude histograms of some traces, suggesting single-channel activity. The conductances indicated are approximate values emerging from a GHK fit of the data. e), f), g): patches from three different cells expressing bTRPV3. Large channel events are clearly visible in NaCl bath solution both at negative potentials (reflecting influx of Na^+^ through channels into the pipette) and at positive potentials (reflecting efflux of NH_4_^+^). h) IV-Plot of the single-channel currents in (e (○), f(□) and g (△)), jointly fitted to the Goldman-Hodgkin-Katz equation and yielding the conductance indicated.

Patches from 21 bTRPV3 cells and 12 control cells were investigated using the inside-out configuration of the patch-clamp technique using an NH_4_Cl pipette solution. At the single-channel level, patches were strikingly different. Only three patches from control cells showed single-channel events in amplitude histograms, yielding a mean conductance of 40.8 ± 11.9 pS for NH_4_^+^ and 25.0 ± 5.8 pS for Na^+^ (n = 3). Conductances in the remaining nine control patches were sometimes considerable (Fig 6B), but could not be resolved in amplitude histograms. In some cases, Ca^2+^ had blocking effects, suggesting expression of a large number of channels with a very low unitary conductance.

**Fig 7:**
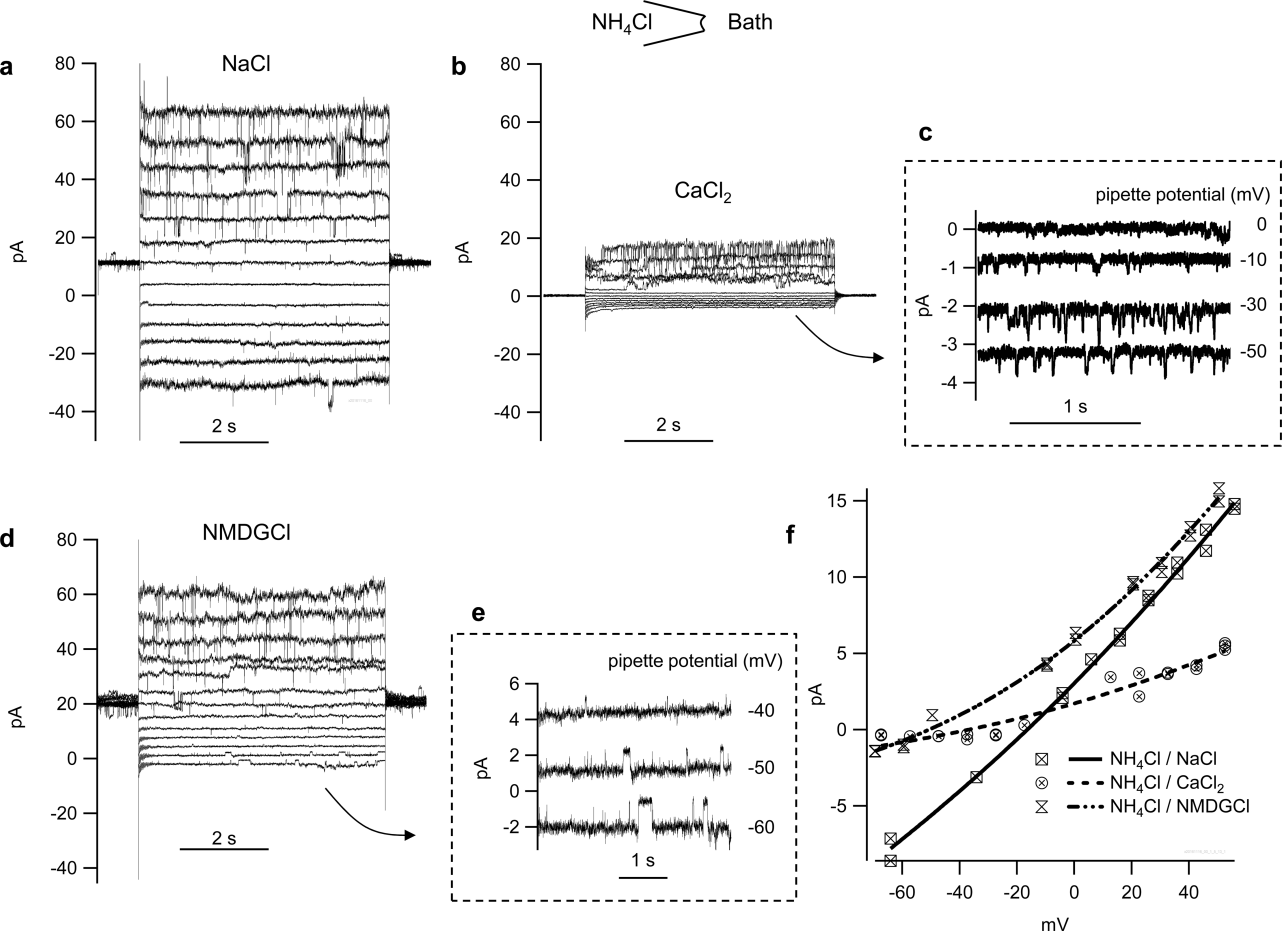
Original recordings of one patch from a bTRPV3 cell with NH_4_Cl in the pipette. a) Channel events are clearly visible in NaCl bath solution in response to pulse protocol III both at negative potentials and positive potentials, reflecting influx of Na^+^ and efflux of NH_4_^+^. b) same patch in 72.5 mmol∙l^−1^ CaCl_2_ bath solution. Ca^2+^ had a clear blocking effect on the current level at positive potentials (efflux of NH_4_^+^). c) Detail from (b), showing single-channel events at negative potentials, reflecting influx of Ca^2+^ d) same patch in NMDGCl bath solution. e) Insert showing single-channel events at negative potential level, suggesting influx of NMDG^+^. f) Current-voltage plot of unitary current amplitudes from this individual patch, fitted with the current formulation of the Goldman-Hodgkin-Katz equation. The fit yields a conductance for Na^+^ of 132 ± 4 pS, for Ca^2+^ of 21 ± 3 pS, and for NMDG^+^ of 36 ± 3 pS. The conductance for NH_4_^+^ was similar in NaCl and NMDGCl solution (250 ± 3 pS or 264 ± 3 pS, respectively) but dropped to 88 ± 4 pS with high Ca^2+^ in the bath.

**Fig 8:**
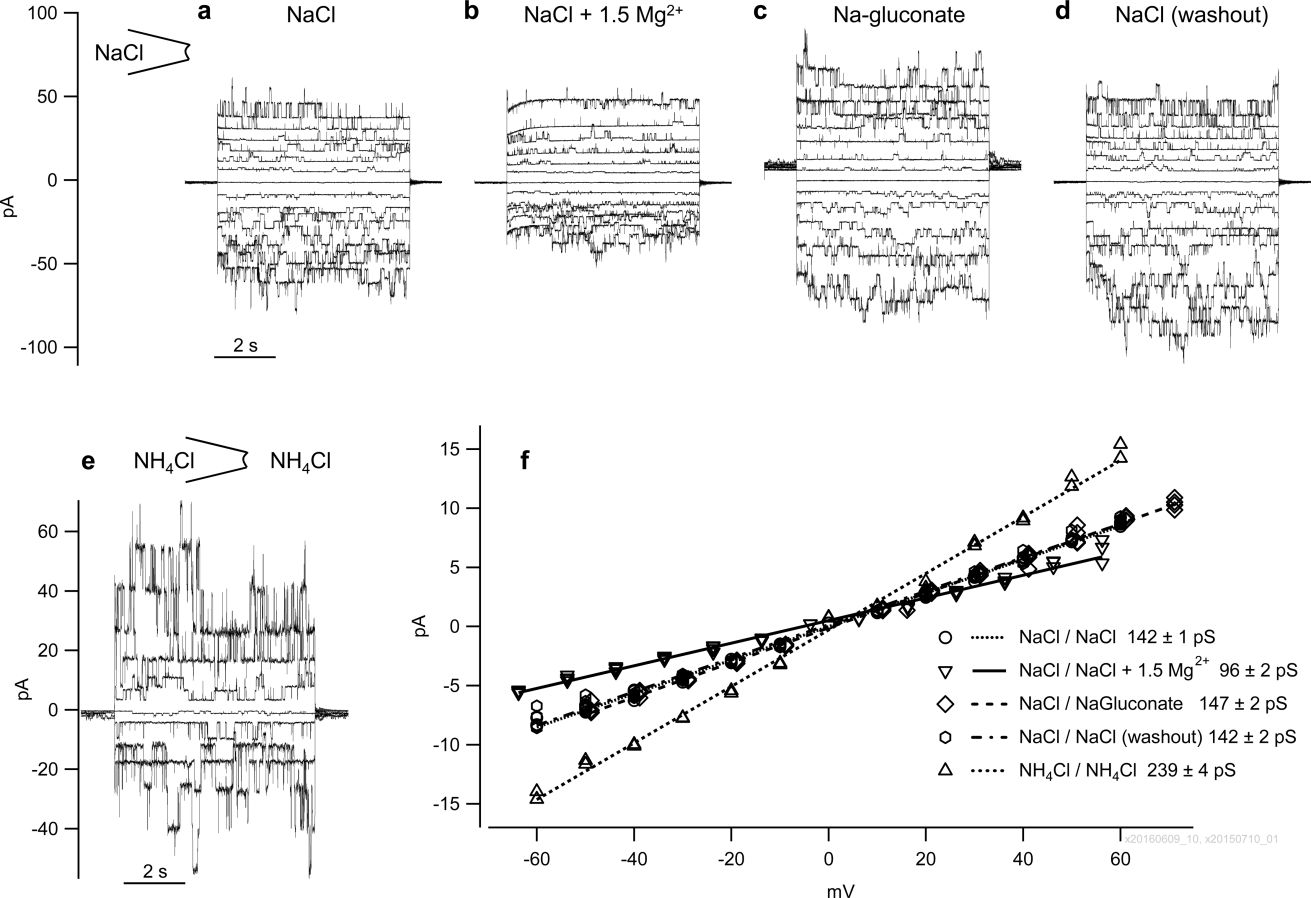
Inside-out measurements from cells expressing bTRPV3 in symmetrical solutions. Panels (a to d) show recordings from the same patch and identical scaling in a) symmetrical NaCl solution, b) NaCl solution with 1.5 mmol∙l ^−1^ MgCl_2_, c) Na-gluconate solution without MgCl_2_ and d) return to symmetrical NaCl solution. The shift in the baseline at 0 mV pipette potential in (c) reflects the impact of the liquid junction potential. e) Patch from a second cell in symmetrical NH_4_Cl solution. For clarity, this graph only shows currents at every other voltage step of pulse protocol III. f) Current-voltage plot of the unitary current amplitudes of the data from (a to e) (linear fit).

Conversely, 19 of 21 patches from bTRPV3 cells showed conductances of over 100 pS, with a Goldman-Hodgkin-Katz fit of the data yielding mean values of 127.1 ± 7.9 pS and 223.0 ± 9.3 pS for Na^+^ and NH_4_^+^, respectively (Fig 6E to 6G and Fig 7A). When the bath solution was switched to NH_4_Cl, a linear IV-relationship emerged with a slope of 240.1 ± 3.6 pS (n = 7) (Fig 8F). In a pure 72.5 mmol∙l^−1^ CaCl_2_ bath solution, Ca^2+^ inhibited the efflux of NH_4_^+^ at positive potentials with conductance dropping significantly to 54.4 ± 13.3 pS (n = 8, p < 0.001, Fig 7F). At negative potentials, channel events reflecting an influx of Ca^2+^ could be observed (Fig 7B and 7C). GHK fits of the data yielded a conductance of 34.0 ± 1.7 pS for Ca^2+^ at 72.5 mmol∙l^−1^ (Fig 7F). Normalized to the concentration, this yields p(Ca^2+^)/p(Na^+^) = [34pS/72.5mmol∙l^−1^]/ [127pS/145mmol∙l^−1^] ≈ 0.53.

In one patch exposed to NMDGCl bath solution, clear channel activity emerged at negative potential levels, suggesting influx of NMDG^+^ (~36 pS) (Fig 7D, 7E and 7F). In two bTRPV3 patches, single-channel events with much lower conductances (39 pS and 73 pS for Na^+^ and 44 pS and 67 pS for NH_4_^+^) were observed. These lower conductances were judged to be endogenous non-selective channels and not included in the averages given above.

#### NH_4_-gluconate pipette solution

A further 15 bTRPV3 inside-out patches were investigated using an NH_4_-gluconate pipette solution, from which 8 seals were stable enough to allow the consecutive application of NH_4_-gluconate, NaCl and NMDG-gluconate in the bath. In symmetrical NH_4_-gluconate solution, the IV-curve of the unitary currents was nonlinear with a conductance of 224.6 ± 5.4 pS at positive potentials (p = 0.06 versus the value in symmetrical NH_4_Cl solution), while at negative potentials, a significantly lower conductance of 180.4 ± 4.2 pS was obtained (p < 0.001). When the bath solution was switched to NaCl, the conductance at positive potentials (reflecting efflux of NH_4_^+^) was 218.2 ± 5.4 pS. This value was not significantly different from that measured in the presence of gluconate (p = 0.328), arguing against a sizable influx of Cl^−^. At negative potentials, a conductance of 151.6 ± 4.8 pS was measured, reflecting influx of Na^+^. This value was significantly higher than that obtained in the previous series of experiments (p = 0.01). The reason for the nonlinear IV-curve in symmetrical NH_4_-gluconate solution and the shift in permeability towards Na^+^ is unclear, but interestingly, changes in bTRPV3 channel characteristics after replacement of chloride by gluconate have also been observed by others [39].

When NMDG-gluconate was applied, no channel events were observed at –60 mV. However, there was sufficient channel activity at lower negative potentials in four patches to allow a GHK fit of the IV-curve, yielding a conductance of 256.5 ± 10.0 pS for the efflux of NH_4_^+^ and of 48.1 ± 3.6 pS for influx of NMDG^+^. Using the value for the conductance to Na^+^ obtained above (151.6 pS), a relative permeability of p(Na^+^)/p(NMDG^+^) ~ 3.2 is obtained.

#### NaCl pipette solution

In symmetrical NaCl solution, a single-channel conductance of 127.9 ± 4.2 pS (n = 20) was measured (Fig 8A to 8D). This value did not differ from p(Na^+^) measured in the asymetrical configuration with NH_4_Cl in the pipette (p = 0.5). Two patches showed the typical bTRPV3 conductance in the range around 125 pS in addition to conductances < 40 pS. These smaller conductances possibly reflected the expression of endogenous channels and were thus not included in the average. Replacement of NaCl by Na-gluconate had no significant impact on conductance (n = 6, p = 0.9). A switch to NH_4_Cl resulted in a conductance to Na^+^ of 140.7 ± 17.7 pS and a conductance to NH_4_^+^ of 211.6 ± 19.6 pS (n = 7, p = 0.016), essentially reflecting the conductances measured with an opposite distribution of ions across the membrane (p > 0.5).

Application of 1.5 mmol∙l^−1^ Mg^2+^ to the NaCl bath solution significantly reduced single-channel conductance for Na^+^ to 81.4 ± 10.2 pS (n = 8, p = 0.047) with a return to the original conductance level after washout. In two patches studied, a similar decrease in conductance was observed after application of 1.5 mmol∙l^−1^ Ca^2+^. Any sign of rectification was very discrete. Times for washout were typically longer than those observed after extracellular exposure of divalent cations.

Although it sometimes appeared as if an exposure to solutions containing Ca^2+^ or Mg^2+^ “triggered” a burst in channel activity, effects were not reversible. Since in general, open probability tended to increase over time, it was not possible to assess the impact of divalent cations on open probability.

#### Changes in conductance and open probability

**Fig 9:**
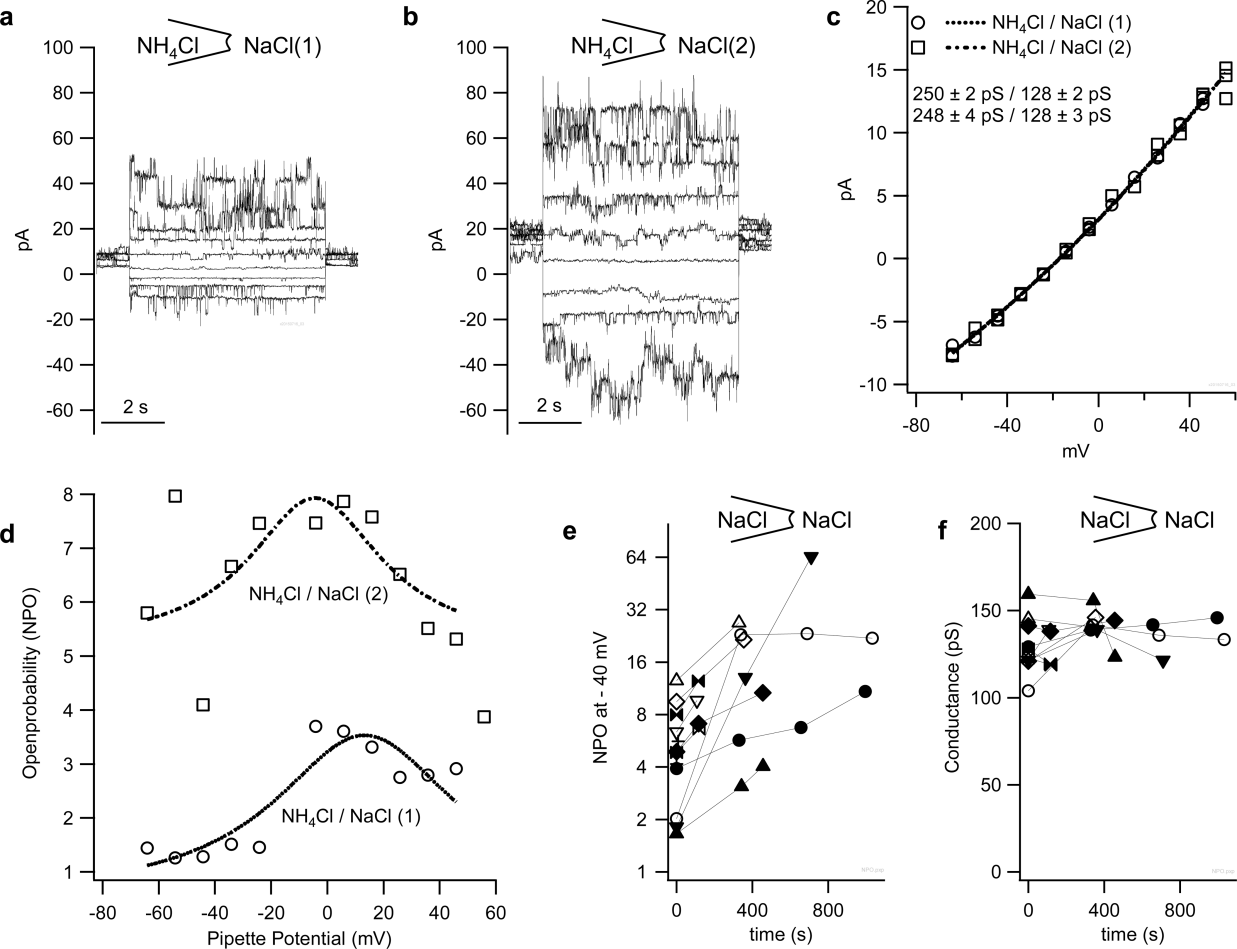
Effects of time after excision on NPo and single-channel conductance. a) Inside-out measurements from a further patch from a cell expressing bTRPV3 with NH_4_Cl in the pipette and NaCl in the bath (shown at −60, −40, −20, −10, 0, 10, 30, and 50 mV). b) same patch in NaCl solution, 100 s later. c) current-voltage plot of unitary currents of the measurements in (a and b). d) Open probability (NPo) of the traces in (a and b) at various voltages. e) The NPo at −40 mV of ten different patches in symmetrical NaCl solution are plotted over the time, beginning with the first trace with channel openings in the amplitude histogram and ending with rupture of the seal (p < 0.001 for consecutive measurements). For optimal representation of low and high values, a logarithmic scaling was chosen. f) Corresponding single-channel amplitude of these ten patches calculated at the time points shown in (e) (no significant change over time, p = 0.9 for consecutive measurements).

At the very beginning of the experiment, open probability was frequently very low, making it difficult to measure single-channel conductance. In most patches, open probability rose with the duration of the experiment, albeit to a highly variable degree (Fig 9D and 9E). Open probability was also slightly voltage-dependent with values higher around 0 mV. In long experiments with multiple solution changes, channel conductance remained the same after a return to the original solution and no indication of a time-dependent “pore dilation” could be observed (Fig 9A–9C).

### Measurements of intracellular Ca^2+^

**Fig 10:**
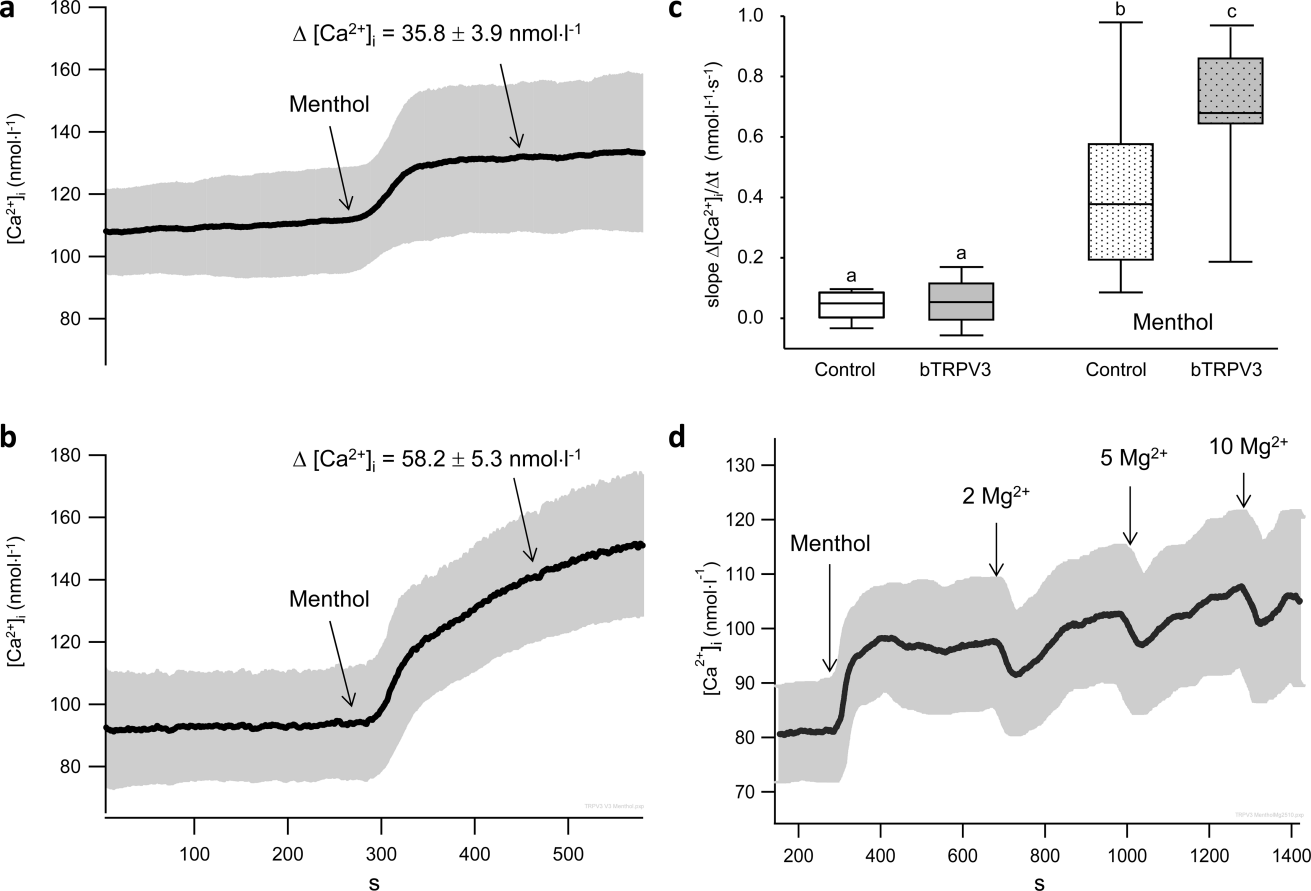
Effects of menthol and Mg^2+^ on intracellular calcium [Ca^2+^]_i_. a) Average of ten original recordings of control HEK-293 cells, showing a significant increase of Δ [Ca^2+^]_i_ in the 200 s interval after application of menthol (1 mmol∙l^−1^) (95% confidence interval in grey), with the timeline identical to that in b), showing the average [Ca^2+^]_i_ (± 95%) of eleven recordings of HEK-293 cells overexpressing bTRPV3. While resting levels of [Ca^2+^]_i_ in bTRPV3 cells were not significantly different from those of control cells, menthol led to a significantly higher Δ [Ca^2+^]_i_ in these cells (p = 0.003). c) Boxplots comparing the slopes (Δ [Ca^2+^]_i_ /Δ t) of the graphs in (a and b) in a 50 s interval at the beginning of the measurement and in the 50 s interval after application of menthol (differing letters above the boxes indicate p < 0.05). d) Average [Ca^2+^]_i_ (± 95%) of further ten recordings of HEK-293 cells overexpressing bTRPV3. Application of 2, 5, and 10 mmol∙l^−1^ Mg^2+^ induced a transient drop in [Ca^2+^]_i_.

In a final series of experiments, the effect of menthol (1 mmol∙l^−1^) on intracellular Ca^2+^ ([Ca ^2+^]_i_) was studied using Fura-2 (Fig 10). Resting levels of [Ca^2+^]_i_ were identical in both groups, but application of menthol induced a significantly higher rise in cells overexpressing bTRPV3 than in controls. The slope in the 50 second interval after application of menthol was also significantly larger in the bTRPV3 expressing cells.

Both when applied before application of menthol and afterwards, extracellular application of Mg^2+^ to bTRPV3 expressing cells induced an immediate reduction of [Ca ^2+^]_i_, with rapid recovery to the original level (Fig 10D).

## Discussion

To the best of our knowledge, this is the first study to rigorously investigate the permeability of a member of the TRP channel family to the physiologically important ammonium ion (NH_4_^+^). We report that in symmetrical NH_4_Cl solution the bovine homologue of TRPV3 conducts ammonia with a single-channel conductance of 240.1 ± 3.6 pS. Single-channel conductance for Na^+^ was 127.9 ± 4.2 pS, that for Ca^2+^ 34.0 ± 1.7 pS. As reported for the human homologue [32], menthol, thymol, carvacrol, and 2-APB stimulated the conductance for monovalent cations, including Na^+^, Cs^+^, K^+^, and NH_4_^+^. In Fura-2 experiments, menthol was additionally shown to increase the uptake of Ca^2+^. Both Ca^2+^ and Mg^2+^ blocked the conductance for monovalent cations. These characteristics precisely fit the profile of the long sought for non-selective cation channel of the ruminal epithelium which has functionally been shown to mediate the absorption of physiologically important cations such as Na^+^, Ca^2+^, and NH_4_^+^ across intact tissues and isolated cells from the native ruminal epithelium *in vitro* [13, 26, 27, 40].

### HEK-293 cells express endogenous small conductance channels permeable to NH_4_^+^

The transport of NH_4_^+^ by K^+^-selective and non-selective cation channels has been observed in a number of preparations in the past [41–43] and from the start we expected a background of endogenous channels to contribute to transport of NH_4_^+^ in control cells. One clear result of our study is that HEK-293 cells also express cation channels permeable to NH_4_^+^, albeit with a much lower single-channel conductance than bTRPV3 cells. On the single-channel level, HEK-293 cells have been shown to express several conductances under 50 pS [37, 44], most likely reflecting expression of non-selective cation channels such as TRPM4 [44] and members of the TRPC family [45] in addition to a plethora of small-conductance K^+^ channels [46, 47]. Probably due to these endogenous channels, differences between HEK-293 cells expressing bTRPV3 and the empty vector were relatively discrete on the whole cell level, except when agonists were applied. Menthol, thymol, carvacrol, and 2-APB enhanced whole cell currents of bTRPV3 expressing HEK-293 cells in striking contrast to the absence of any effect in controls (Fig 4 and Table 3). This result confirms previous reports that any endogenous expression of TRPM8 or TRPV3 by HEK-293 cells is very small [48, 49]. In cells filled with K-gluconate solution, menthol and particularly 2-APB also increased the influx of NH_4_^+^ and the efflux of K^+^ at negative and positive potentials, respectively.

### Expression of bTRPV3 induces a large single-channel conductance to Na^+^ and NH_4_^+^

Impressive differences between control and bTRPV3 cells emerged when patches were investigated on the single-channel level. Nine out of ten patches from bTRPV3 expressing cells showed large single-channel conductances with a permeability of 240 pS for NH_4_^+^ and 130 pS for Na^+^. Interestingly, linear fits from symmetrical configurations yielded similar values as GHK fits from asymmetrical configurations. This not only confirms the reproducibility of our data but suggests that NH_4_^+^ and Na^+^ do not interfere with each other in the channel pore [50] with important implications for physiological situations with low NH_4_^+^ and high Na^+^ concentration. In contrast, deviations from the independence principle were observed in configurations where either cation interacted with Ca^2+^, Mg^2+^, or NMDG^+^.

The value obtained for the conductance of Na^+^ under symmetrical conditions in our experiments was significantly lower than the 190 pS reported for the human homologue under conditions identical to this study (145 mmol∙l^-1^ symmetrical NaCl, room temperature) [33]. However, it should be noted that Doerner et al. pretreated excised patches with PI(4,5)P_2_-depleting agents and diC8-PI(4,5)P_2_. Xu et al. [35] measured a slightly lower conductance of 172 pS using a Cs-methane-gluconate pipette solution in NaCl bath. Human TRPV3 protein and the bovine homologue used in this study show a high similarity (96.8%) and align perfectly in the pore region, including the filter [51] (see S1 File). The pore region can therefore be ruled out as a reason for this difference. Likewise, the two residues (His^426^ and Arg^696^) specifically required for sensitivity of TRPV3 to 2-APB [52] are conserved between hTRPV3 and bTRPV3. Differences emerge in a key part of a calmodulin binding domain between Arg^113^ and Arg^122^ [39, 53], which reads RRKKRRLKKR in *Homo sapiens*, RQKKKRLKKR in *Mus musculus*, and RRKKKRLKKR in the *Bos taurus* sequence of this study (difference versus *Homo sapiens* underlined). Depending on how much Ca^2+^ is bound, this cytosolic N-terminal region folds to obstruct the pore region, modulating conductance. Intriguingly, studies of the murine homologue of TRPV3 report a lower conductances of 147 pS for Na^+^ at positive potentials [54] and a lower value of 101 pS for Cs^+^ in the cell-attached configuration [55].

### Menthol and the regulation of [Ca^2+^]_i_

The functional downregulation of Ca^2+^-permeable TRP channels by Ca^2+^-calmodulin generally serves as an important negative feedback mechanism to prevent excessive Ca^2+^ influx into cells, preventing toxicity [56] (see S1 Fig). Indeed, resting intracellular Ca^2+^ levels in bTRPV3 cells were maintained at levels comparable to those found in the control cells (Fig 10). Regulation of channel conductance and open probability by this cytosolic Ca^2+^-calmodulin complex might also explain the transient nature of the Mg^2+^-induced drop in [Ca^2+^]_i_ observed via ratiometric calcium imaging (Fig 10D): after a drop in Ca^2+^ influx (e.g. after extracellular application of Mg^2+^ with obstruction of the TRPV3 pore via interaction with the channel pore [53, 57]), the Ca^2+^-calmodulin gate should reopen until [Ca^2+^]_i_ returns to the initial “set” value, as observed. In contrast to the transient effects of Mg^2+^, the menthol-induced response persisted. Interestingly, in TRPM8, menthol has been shown to slow down channel gating by stabilizing the open state [58] but it is unclear if this stabilization involves effects on the calmodulin gate. In TRPA1, menthol interacts with transmembrane domain 5 [59]. However, TM5 of TRPA1 does not align with that of TRPV3 and the ligand binding sites responsible for menthol effects on TRPV3 remain to be identified [60].

The effect of menthol on [Ca^2+^]_i_ of control HEK-293 cells was unexpected but has been reported previously [49] and probably reflects a release of [Ca^2+^]_i_ from intracellular stores unrelated to expression of membrane channels. Notably, ratiometric calcium imaging was carried out at 37°C, where this pathway is active, whereas at the 23°C used in our patch-clamp experiments, it is inactivated.

### Single-channel conductance of bTRPV3 to Ca^2+^

This is also the first time that the single-channel conductance for Ca^2+^ has been directly measured for a TRPV3 homologue. As to be expected, the conductance of the channel for Ca^2+^ (hydration energy: −1579 kJ∙mol^−1^) was lower than that for Na^+^ (−406 kJ∙mol^−1^). From the single-channel conductance and normalized to concentration, we report a value of p(Ca^2+^)/p(Na^+^) of ~ 0.5. However, this value is strikingly smaller than the generally quoted value of 10 to 12 [35, 57]. Notably, these “classical” values were not obtained from single-channel measurements but from shifts in reversal potential measured in the whole cell configuration with a Cs-gluconate or -aspartate pipette solution and an NMDGCl or NaCl bath, to which Ca^2+^ was added in concentrations of 5 or 30 mmol∙l^−1^. In this configuration, addition of Ca^2+^ blocked Cs^+^ efflux and led to a shift in reversal potential that is entirely unrelated to the permeability of bTRPV3 to Ca^2+^. A systematic overestimation of p(Ca^2+^)/p(Na^+^) is therefore to be expected. To avoid this problem, we used symmetrical Na- and NMDG-gluconate solutions in our whole cell experiments, in which changes in the permeability of the monovalent cation will not result in a shift of the reversal potential. Accordingly, our values for p(Ca^2+^)/p(Na^+^) from whole cell experiments were much lower than those in the literature and similar to those obtained via direct measurements of single-channel activity. However, the application of Goldman-Hodgkin-Katz theory to a situation where the independence principle clearly does not hold remains problematic [28].

### Interaction with divalent cations

As observed in other studies of TRPV3 [39, 53, 61] as well as in the intact ruminal tissue [13, 26], divalent cations such as Ca^2+^ and Mg^2+^ inhibit monovalent currents (Table 1, Figs 2 and 3). Aspartate residues in the filter region (Asp^641^) of TRPV3 have been implicated in what appears to be a direct and voltage-dependent interaction of extracellular divalent cations with the channel conductance [53] (see S1 File and S1 Fig). In NMDG solutions, the current kinetics became clearly visible (Fig 5), showing how at positive potentials the blocking effects of divalent cations decreased over time whereas at negative potentials the block increased with time. Most likely, this reflects an open pore blocking mechanism. Accordingly, a positive voltage pulse leads to a gradual relief of the block whereas a negative pipette potential serves as a driving force for the insertion of blocking divalent cations into the channel pore. Conversely, when Ca^2+^ or Mg^2+^ were intracellularly applied, unitary conductance dropped but no obvious sign of rectification could be observed (Fig 8F). The reason for this may be that on the cytoplasmic side, Ca^2+^ does not interact directly with the pore region but binds to the previously mentioned N-terminal calmodulin-binding site, which then obstructs the pore region [39, 53]. Chemical or physical stimuli–such as patch excision–can interfere with this mechanism, sensitize TRPV3, and decrease the voltage-dependence of a block by cytosolic Ca^2+^ [53].

The voltage-dependent block of TRPV3 by divalent cations may have consequences for understanding the regulation of Na^+^ transport across the rumen *in vivo*. Thus, it has long been known that when ruminants are switched from a (winter) diet of hay to spring grass, a rise in intraruminal K^+^ concentration occurs that can exceed 100 mmol∙l^-1^ [62, 63]. A concomitant depolarization of the epithelium is observed *in vivo* [62, 64] and *in vitro* [65], associated with an increase in the uptake of Na^+^, so that the sum of the two cations is maintained at a constant level. *In vitro* experiments in the Ussing chamber suggest that this increase in Na^+^ absorption is related to the opening of a voltage-dependent cation channel in the apical membrane of the epithelium [40]. Although lacking a classical voltage sensor, TRPV3 appears as a suitable candidate. When the apical membrane is depolarised (e.g. by application of a potential *in vitro* or high K^+^ *in vivo*), the divalent cations that normally block the pore are repelled, resulting in an increased permeation of monovalent cations such as Na^+^. Accordingly, ruminal osmolarity is maintained even when dramatic changes in the K^+^ concentration of the diet occur. It may be mentioned that in these feeding situations clinical problems can occur related to a decrease in the absorption of Mg^2+^ (most likely via TRPM6 [13] and TRPM7 [9]).

### A conductance for NMDG^+^?

Increasing physical disruption of the interaction of the Ca^2+^-calmodulin complex with the pore region may also be one reason why after excision, the open probability of bTRPV3 channels typically increased, coupled to a decrease in the small intrinsic voltage-dependence of the channel in Ca^2+^-free solution (Fig 9D). Most likely, the increase in open probability at negative potential levels was also the major factor leading to an increased influx of NMDG^+^ (Figs 5D and 7D). Since the single-channel conductance for Na^+^ in Ca^2+^-free solution did not change during our experiments (Fig 9C), we would tend to argue that the conductance to NMDG^+^ was not a result of “pore dilation” [57, 66].

Notably, Doerner et al. also specifically reported having observed certain cells with an inward current in NMDGCl solution [33]. Interestingly, our value of p(Na^+^)/p(NMDG^+^) of ~ 2.7 and 3.2 –obtained within the first 300 s from whole cell and single-channel data at room temperature–corresponds reasonably well with the value reported by Chung et al. [57] ~ of 2.9 from whole cell experiments on murine TRPV3-HEK-293 cells after a heat stimulus.

### A role for bTRPV3 in ruminal transport

In the current study, we demonstrate that HEK-293 cells overexpressing bTRPV3 share the pharmacological properties observed in studies of Na^+^, K^+^, Ca^2+^, and NH_4_^+^ transport across intact tissues and cells isolated from the native ruminal epithelium [11, 13, 25, 27, 40]. In conjunction with the high expression levels of mRNA found in this tissue and the observation that TRPV3 agonists stimulate the transport of Na^+^, NH_4_^+^ and Ca^2+^ across the tissue *in vitro* [13], bTRPV3 emerges as a main contributor to the divalent sensitive, non-selective cation conductance previously described [26, 27, 40]. However, it appears very likely that further (TRP-) channels are involved. Given that any ruminal expression of the classical epithelial calcium channels TRPV5 or TRPV6 is marginal at best [13, 67], bTRPV3 also appears as a suitable candidate for mediating ruminal Ca^2+^ absorption, which is essential for maintaining Ca^2+^ homeostasis in lactating animals. Our observation that Mg^2+^ negatively interacts with uptake of Ca^2+^ via bTRPV3 should be considered when discussing the interactions of high amounts of dietary Mg^2+^ with Ca^2+^ homeostasis in ruminants [68].

The finding that bTRPV3 can transport NH_4_^+^ or possibly even larger cations such as NMDG^+^ is novel and can help to explain why losses of ammonia from the rumen are so high even in feeding situations where ruminal pH is low. At first glance, loss of NH_4_^+^ from the rumen via bTRPV3 would appear to be detrimental to the animal, since the availability of nitrogen for conversion to bacterial protein is reduced. However, bTRPV3 may contribute to ruminal pH homeostasis, in particular in conjunction with urea recycling as discussed above [20, 21]. Furthermore, a shift away from the absorption of (alkaline) NH_3_ towards NH_4_^+^ will decrease the pH of the portal blood and induce an activation of glutamine synthetase. This, in turn, will increase the hepatic production of glutamine and reduce the production of urea [69]. Glutamine is not only an important source of energy for rapidly dividing cells, but can also be utilized by the liver for the synthesis of other non-essential amino-acids. In catabolic states, plasma levels of glutamine frequently drop considerably so that mobilization from muscle stores is required, as around calving in ruminants [70]. Ultimately, it appears likely that a fine balance has to be maintained between retention of ammonia in the rumen and absorption either in the form of NH_3_ or NH_4_^+^. Ruminants are known to be selective grazers with a preference for certain plants [71], and it is intriguing to speculate on the role that selective uptake of herbal compounds with action on TRPV3 may play for the health of free-ranging animals in habitats with a diverse vegetation.

Given that ammonia is a ubiquitous by-product of protein metabolism, other implications follow and the permeability of TRPV3 to NH_4_^+^ should be considered when discussing the function of this poorly understood channel in other tissues, such as in the colon [72] or the skin [29].

## Supporting information

S1 Fig Cartoon of TRPV3, depicting the six membrane-spanning domains with the pore region between TM5 and TM6 [39, 52, 53, 55, 73]. Four of the depicted units associate to form a channel. Extracellular Ca^2+^ interferes with the permeation of monovalent cations by binding to the filter region via a voltage dependent mechanism, with depolarization “kicking out” the divalent cation and thus alleviating the block. Mg^2+^ block is similar and may also involve the same region. Intracellular Ca^2+^ modulates channel activity by binding to a calmodulin domain that is found N-terminally. In addition, Ca^2+^ modulates channel activation by 2-aminoethoxydiphenylboronate (2-APB).
(TIF)

S1 File Alignment of Mus musculus Trpv3 (NM_145099), Bos taurus TRPV3 (XM_015458625.1) and Homo sapiens TRPV3 (NM_001258205.1). Conserved amino acid residues are marked with an (*). Functionally important regions are color-coded as described below.
(DOCX)
