# Supplementary figures and images for: The bovine TRPV3 as a pathway for the uptake of Na+, Ca2+, and NH4+
Source: PLoS One. 2018 Mar 1;13(3):e0193519. doi: 10.1371/journal.pone.0193519 (PMC5832270; doi:10.1371/journal.pone.0193519)

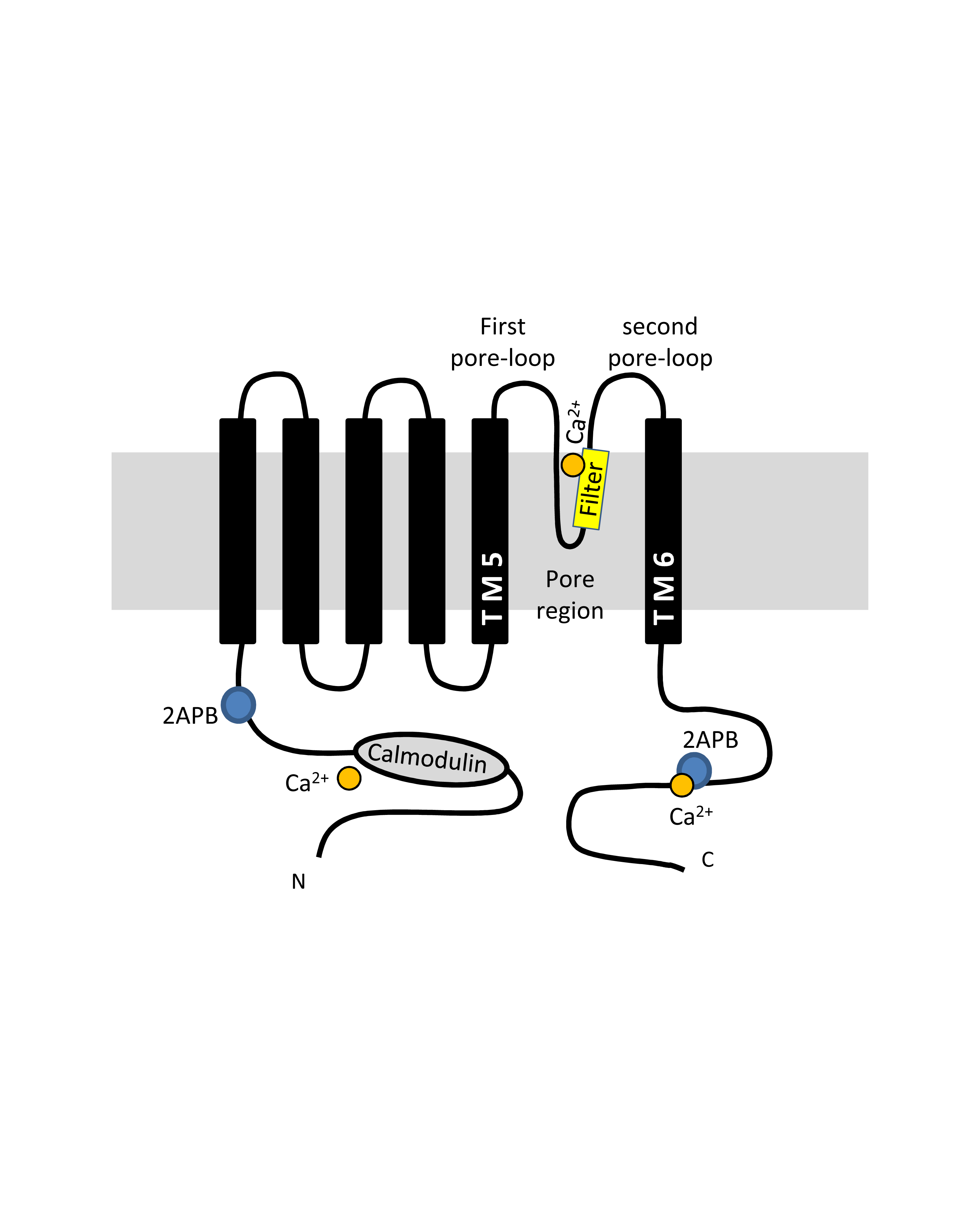

Supplement: S1 Fig — Four of the depicted units associate to form a channel. Extracellular Ca2+ interferes with the permeation of monovalent cations by binding to the filter region via a voltage dependent mechanism, with depolarization “kicking out” the divalent cation and thus alleviating the block. Mg2+ block is similar and may also involve the same region. Intracellular Ca2+ modulates channel activity by binding to a calmodulin domain that is found N-terminally. In addition, Ca2+ modulates channel activation by 2-aminoethoxydiphenylboronate (2-APB). (TIF) [file pone.0193519.s001.tif]
