# Supplementary material for: The bovine TRPV3 as a pathway for the uptake of Na+, Ca2+, and NH4+
Source: PLoS One. 2018 Mar 1;13(3):e0193519. doi: 10.1371/journal.pone.0193519 (PMC5832270; doi:10.1371/journal.pone.0193519)
Supplement: S1 File — Conserved amino acid residues are marked with an (*). Functionally important regions are color-coded as described below. (DOCX) [file pone.0193519.s002.docx]

**S1 File. Alignment of Mus musculus Trpv3 (NM_145099), Bos taurus TRPV3 (XM_015458625.1) and Homo sapiens TRPV3 (NM_001258205.1).** Conserved amino acid residues are marked with an (*). Functionally important regions are color-coded as described below.

Reference

DAVLELFKLTI Pore region [[1](#_ENREF_1)]

GLGDL Filter region [[2](#_ENREF_2)]

FLFVYILFLLGFGVALASLIEK TM5 [[2](#_ENREF_2)]

CSKDKKDCSSYGSFS First pore-loop [[2](#_ENREF_2)]

NIQQNSTYPIL Second pore-loop [[2](#_ENREF_2)]

FLFLLITYVILTFVLLLN TM6 [[2](#_ENREF_2)]

*H,* ***R*** APB sensitive [[3](#_ENREF_3)]

**D** extracellular Ca2+ binding site [[4](#_ENREF_4)]

***R*** intracellular Ca2+ binding site [[3](#_ENREF_3)]

AKEEQ*RQKKKRLKKR* Calmodulin binding site [[4](#_ENREF_4), [5](#_ENREF_5)]

mTRPV3 -------MNAHSKEMAPLMGKRTTAPGGNPVVLTEKRPADLTPTKKSAHFFLEIEGFEPN 53

bTRPV3 MSLCRTAMKAHPKEMVPLTGRRATIPFVNPAIMQEKRPSEITPTKKSAHFFLEIEGFEPN 60

hTRPV3 -------MKAHPKEMVPLMGKRVAAPSGNPAILPEKRPAEITPTKKSAHFFLEIEGFEPN 53

*:** ***.** *:*.: * **.:: ****:::*******************

mTRPV3 PTVTKTSPPIFSKPMDSNIRQCLSGNCDDMDSPQSPQDDVTETPSNPNSPSANLAKEEQ*R* 113

bTRPV3 PTVAKTSPPIFSKPMDSNIRQCVSGNCDDMDSPQSPQDDVTETPSNPNSPSANLAKEEQ*R* 120

hTRPV3 PTVAKTSPPVFSKPMDSNIRQCISGNCDDMDSPQSPQDDVTETPSNPNSPSAQLAKEEQ*R* 113

***:*****:************:*****************************:*******

mTRPV3 *QKKKRLKKR*IFAAVSEGCVEELRELLQDLQDLCRRRRGLDVPDFLMHKLTASDTGKTCLM 173

bTRPV3 *RKKKRLKKR*IFTAVSEGCVEELLELLGELQELCKRRHSLDVPDFLMHKLTALDTGKTCLM 180

hTRPV3 *RKKRRLKKR*IFAAVSEGCVEELVELLVELQELCRRRHDEDVPDFLMHKLTASDTGKTCLM 173

:**:*******:********** *** :**:**:**:. ************ ********

mTRPV3 KALLNINPNTKEIVRILLAFAEENDILDRFINAEYTEEAYEGQTALNIAIERRQGDITAV 233

bTRPV3 KALLNINPNTKEIVRILLAFAEENDILDRFINAEYTEEAYEGQTALNIAIERRQGDITAA 240

hTRPV3 KALLNINPNTKEIVRILLAFAEENDILGRFINAEYTEEAYEGQTALNIAIERRQGDIAAL 233

***************************.*****************************:*

mTRPV3 LIAAGADVNAHAKGVFFNPKYQHEGFYFGETPLALAACTNQPEIVQLLMENEQTDITSQD 293

bTRPV3 LIAAGADVNAHAKGVFFNPKYQHEGFYFGETPLALAACTNQPEIVQMLMENEQTDITSQD 300

hTRPV3 LIAAGADVNAHAKGAFFNPKYQHEGFYFGETPLALAACTNQPEIVQLLMEHEQTDITSRD 293

**************.*******************************:***:*******:*

mTRPV3 SRGNNILHALVTVAEDFKTQNDFVKRMYDMILLRSGNWELETMRNNDGLTPLQLAAKMGK 353

bTRPV3 SRGNNILHALVTVAEDFKTQNDFVKRMYDMILLRSRTWELETTRNNDGLTPLQLAAKMGK 360

hTRPV3 SRGNNILHALVTVAEDFKTQNDFVKRMYDMILLRSGNWELETTRNNDGLTPLQLAAKMGK 353

*********************************** .***** *****************

mTRPV3 AEILKYILSREIKEKPLRSLSRKFTDWAYGPVSSSLYDLTNVDTTTDNSVLEIIVYNTNI 413

bTRPV3 AEILKYILSREIKDKRLRSLSRKFTDWAYGPVSSSLYDLTNVDTTTDNSVLEIIVYNTNI 420

hTRPV3 AEILKYILSREIKEKRLRSLSRKFTDWAYGPVSSSLYDLTNVDTTTDNSVLEITVYNTNI 413

*************:* ************************************* ******

mTRPV3 DNRHEMLTLEPL*H*TLLHTKWKKFAKYMFFLSFCFYFFYNITLTLVSYYRPREDEDLPHPL 473

bTRPV3 DNRHEMLTLEPL*H*TLLHMKWKKFAKYMFFLSFCFYFFYNITLTLVSYYRPREEEALPHPL 480

hTRPV3 DNRHEMLTLEPL*H*TLLHMKWKKFAKHMFFLSFCFYFFYNITLTLVSYYRPREEEAIPHPL 473

***************** *******:**************************:* :****

mTRPV3 ALTHKMSWLQLLGRMFVLIWATCISVKEGIAIFLLRPSDLQSILSDAWFHFVFFVQAVLV 533

bTRPV3 ALTHKMGWLQLLGRMFVLIWAMFISVKEGIAIFLLRPSDLQSILSDAWFHFVFFAQAVLV 540

hTRPV3 ALTHKMGWLQLLGRMFVLIWAMCISVKEGIAIFLLRPSDLQSILSDAWFHFVFFIQAVLV 533

******.************** ******************************* *****

mTRPV3 ILSVFLYLFAYKEYLACLVLAMALGWANMLYYTRGFQSMGMYSVMIQKVILHDVLKFLFV 593

bTRPV3 ILSVFLYLFAYKEYLACLVLAMALGWANMLYYTRGFQSMGMYSVMIQKVILHDVLKFLFV 600

hTRPV3 ILSVFLYLFAYKEYLACLVLAMALGWANMLYYTRGFQSMGMYSVMIQKVILHDVLKFLFV 593

************************************************************

mTRPV3 YILFLLGFGVALASLIEKCSKDKKDCSSYGSFSDAVLELFKLTIGLG**D**LNIQQNSTYPIL 653

bTRPV3 YIVFLLGFGVALASLIEKCPKSHENCSSYGSFSDAVLELFKLTIGLG**D**LNIQQNSKYPIL 660

hTRPV3 YIVFLLGFGVALASLIEKCPKDNKDCSSYGSFSDAVLELFKLTIGLG**D**LNIQQNSKYPIL 653

**:**************** *.:::******************************.****

mTRPV3 FLFLLITYVILTFVLLLNMLIALMGETVENVSKESERIWRLQ***R***ARTILEFEKMLPEWLRS 713

bTRPV3 FLFLLITYVILTFVLLLNMLIALMGETVENVSKESERIWRLQ***R***ARTILEFEKILPEWLRS 720

hTRPV3 FLFLLITYVILTFVLLLNMLIALMGETVENVSKESERIWRLQ***R***ARTILEFEKMLPEWLRS 713

****************************************************:*******

mTRPV3 RFRMGELCKVADEDFRLCLRINEVKWTEWKTHVSFLNEDPGPIRRTADLNKIQDSSRSNS 773

bTRPV3 RFRMGELCKVAEDDFRLCLRINEVKWTEWKTHVSFLNEDPGPGRRTADSNKIQDSSRSNS 780

hTRPV3 RFRMGELCKVAEDDFRLCLRINEVKWTEWKTHVSFLNEDPGPVRRTADFNKIQDSSRNNS 773

***********::***************************** ***** ********.**

mTRPV3 KTTLYAFDELDEFPETSV 791

bTRPV3 KTTLNAFEEIDEFPETSV 798

hTRPV3 KTTLNAFEEVEEFPETSV 791

**** **:*::*******

**References for the Supplemental File**

1. Grandl J, Hu H, Bandell M, Bursulaya B, Schmidt M, Petrus M, et al. Pore region of TRPV3 ion channel is specifically required for heat activation. Nat Neurosci. 2008;11(9):1007-13. Epub 2009/01/23. doi: 10.1038/nn.2169

nn.2169 [pii]. PubMed PMID: 19160498; PubMed Central PMCID: PMC2685190.

2. Kim SE, Patapoutian A, Grandl J. Single residues in the outer pore of TRPV1 and TRPV3 have temperature-dependent conformations. PLoS One. 2013;8(3):e59593. Epub 2013/04/05. doi: 10.1371/journal.pone.0059593

PONE-D-12-35833 [pii]. PubMed PMID: 23555720; PubMed Central PMCID: PMC3608658.

3. Hu H, Grandl J, Bandell M, Petrus M, Patapoutian A. Two amino acid residues determine 2-APB sensitivity of the ion channels TRPV3 and TRPV4. Proc Natl Acad Sci U S A. 2009;106(5):1626-31. Epub 2009/01/24. doi: 10.1073/pnas.0812209106

0812209106 [pii]. PubMed PMID: 19164517; PubMed Central PMCID: PMC2635798.

4. Xiao R, Tang J, Wang C, Colton CK, Tian J, Zhu MX. Calcium plays a central role in the sensitization of TRPV3 channel to repetitive stimulations. J Biol Chem. 2008;283(10):6162-74. doi: 10.1074/jbc.M706535200. PubMed PMID: 18178557; PubMed Central PMCID: PMC2287377.

5. Phelps CB, Wang RR, Choo SS, Gaudet R. Differential regulation of TRPV1, TRPV3, and TRPV4 sensitivity through a conserved binding site on the ankyrin repeat domain. J Biol Chem. 2010;285(1):731-40. doi: 10.1074/jbc.M109.052548. PubMed PMID: 19864432; PubMed Central PMCID: PMC2804222.
